# Artificial Intelligence in Photodynamic Therapy: From Molecular Design of Photosensitizers to Intelligent Delivery, Adaptive Dosimetry, and Personalized Prognostication of Results

**DOI:** 10.3390/ijms27198871

**Published:** 2026-10-05

**Authors:** Rostyslav Marunych, Dorota Bartusik-Aebisher, Klaudia Dynarowicz, Barbara Smolak, David Aebisher

**Affiliations:** 1 Doctoral School, Faculty of Medicine, Collegium Medicum, University of Rzeszów, 35-310 Rzeszów, Poland; rostyslavm@dokt.ur.edu.pl; 2 Department of Biochemistry and General Chemistry, Faculty of Medicine, Collegium Medicum, University of Rzeszów, 35-310 Rzeszów, Poland; dbartusikaebisher@ur.edu.pl (D.B.-A.); kdynarowicz@ur.edu.pl (K.D.); 3 Department of Diagnostic Imaging and Nuclear Medicine, Faculty of Medicine, University of Rzeszów, 35-310 Rzeszów, Poland; bsmolak@ur.edu.pl; 4 Department of Photomedicine and Physical Chemistry, Faculty of Medicine, Collegium Medicum, University of Rzeszów, 35-959 Rzeszów, Poland

**Keywords:** photodynamic therapy, artificial intelligence, machine learning, photosensitizer, drug delivery, nanomedicine, treatment planning, dosimetry, radiomics, personalized medicine

## Abstract

Photodynamic therapy (PDT) is a minimally invasive therapeutic approach based on the light-induced activation of a photosensitizer in the presence of molecular oxygen, resulting in the generation of reactive oxygen species, including singlet oxygen. Despite local selectivity and low systemic toxicity, reproducibility of PDT is restricted by non-uniform accumulation of the photosensitizer, aggregation and insufficient solubility of compounds, variability in the optical properties of tissues, hypoxia, oxygen consumption, photobleaching, and the lack of a universal measure of photodynamic dose. Artificial intelligence (AI), including machine learning and deep learning, graph neural networks, generative models, Bayesian optimization, and physics-informed neural networks, can integrate molecular, spectral, imaging, dosimetric, omics, and clinical data into decision-support systems. In this review, a closed AI–PDT cycle is considered, which includes the in silico design of photosensitizers, optimization of nanoformulations and delivery, personalized light exposure planning, real-time monitoring of photobleaching and oxygenation, and prediction of efficacy, toxicity, immune response, and long-term outcomes. Particular attention is given to data quality, external validation, interpretability, uncertainty assessment, and clinical readiness. This review emphasizes that the greatest potential of AI in PDT lies in its integration with mechanistic models of photochemistry, light transport, and oxygen dynamics, enabling the transition from empirical treatment protocols toward adaptive, reproducible, and personalized photomedicine. Unlike reviews focused on individual AI applications, this work considers the entire AI–PDT workflow as an interconnected system linking molecular design, drug delivery, dosimetry, treatment monitoring, and outcome prediction, while maintaining clinician oversight and the need for experimental and clinical validation.

## 1. Introduction

### 1.1. Modern Stage of Photodynamic Therapy

Photodynamic therapy is a physicochemical method of local action, in which a photosensitizer that is therapeutically inert or lowly toxic in the dark is activated by light in the presence of molecular oxygen. After absorbing a photon, the photosensitizer molecule transitions from the ground state to an excited singlet state, and then, due to intersystem crossing, can form a longer-lived triplet state.

Its next interaction with biomolecules and oxygen is realized primarily through type I reactions. This involves the transfer of an electron or hydrogen atom with the formation of radical species, or through type II reactions, in which energy is transferred to molecular oxygen with the generation of singlet oxygen. Both mechanisms can occur simultaneously, and their relative contribution is determined by the chemical structure of the photosensitizer, the efficiency of intersystem crossing, the state of aggregation, the polarity and viscosity of the microenvironment, the availability of electron donors, and the local oxygen concentration [1,2,3,4,5].

The biological effect of PDT is formed at several interconnected levels. At the molecular level, reactive oxygen species oxidize unsaturated lipids, amino acid residues of proteins, nucleotides, and low-molecular-weight antioxidants. At the organelle level, mitochondria, lysosomes, the endoplasmic reticulum, the Golgi apparatus, and the plasma membrane are damaged. At the cellular level, the combination of the intensity of oxidative stress, photosensitizer localization, and the cellular energy state determines the process of cell death, as well as the prevalence of apoptosis, necrosis, autophagy-mediated cell death, ferroptosis, or a mixed phenotype. In tumor tissue, direct cytotoxicity is accompanied by vascular damage, impaired perfusion, local inflammation, and immunogenic cell death, which can enhance the presentation of tumor antigens and antitumor immunity [3,4,5,6,7,8,9,10,11,12]. Therefore, PDT cannot be adequately described solely by the amount of light energy delivered: the therapeutic outcome represents a causal chain from the electronic structure of the photosensitizer to the spatiotemporal tissue response and systemic immune response.

The clinical importance of PDT is valued for its minimal invasiveness, the absence of a cumulative ionizing dose, the possibility of repeated administration, the local nature of the effect, and the potentially favorable cosmetic outcome. The method is used, or is being actively studied, in dermatology, oncology, ophthalmology, dentistry, antimicrobial therapy, and interventional procedures [1,3,4]. The development of endoscopic, interstitial, and intraoperative systems is expanding its use beyond superficial lesions. At the same time, the diversity of photosensitizers, light sources, administration routes, and clinical indications has led to the development of numerous protocols that are difficult to compare. Even with the same nominal fluence, the actual energy absorbed and the number of reactive species formed can vary significantly due to variations in absorption and scattering, drug concentration, subcellular localization, blood supply, oxygenation, and photobleaching rate [5,6,7]. This dependence explains why results obtained for one molecule, tumor model, or light setup cannot always be directly transferred to another system.

### 1.2. Main Limitations of Modern PDT

The first group of limitations of modern PDT is related to the properties of photosensitizers. Many porphyrin, chlorin, and phthalocyanine compounds are highly hydrophobic, prone to π–π aggregation, and, when aggregated, exhibit a reduced quantum yield of reactive oxygen species, excited-state quenching, and unpredictable in vivo distribution. Accumulation of the photosensitizer in normal tissues can be caused by atypical selectivity. This can generate an effect of prolonged cutaneous photosensitivity. Furthermore, simply increasing hydrophilicity often impairs membrane transport, while the introduction of a charge, a heavy atom, or a metal coordination, either within the chromophore core or at peripheral positions depending on the photosensitizer class, simultaneously alters intersystem conversion and spectral properties. These facts have an influence on pharmacokinetics and toxicity. The development of a new photosensitizer is a complicated task. It is a multi-criteria task requiring optimization of absorption band positions, molar extinction coefficients, triplet and singlet oxygen quantum yields, solubility, aggregation, biodistribution, organelle localization, dark toxicity, metabolic stability, and synthetic availability [1,2,8,9]. Traditional sequential synthesis of analogs scales poorly to such a large number of interdependent parameters.

The second group of limitations consists of the delivery of the photosensitizer, light, and oxygen. Systemic or local administration does not maintain uniform drug accumulation in the tumor. Distribution and localization depend on the size and charge of the molecule or nanoparticle, protein binding, vascular permeability, interstitial pressure, cellular transport processes, and extracellular matrix density. Light is both absorbed and scattered by biological tissue. This makes superficial modes weakly tolerated by large or deep tumors if the treatment is carried out without interstitial light guides, endoscopic systems, or intraoperative applicators. Non-uniform illumination creates areas of insufficient dose near areas of overexposure and increases the risk of both preserving a viable tumor and damaging critical normal structures.

Tumor hypoxia further reduces the effectiveness of oxygen-dependent mechanisms, and intense irradiation can deplete oxygen faster than it is supplied by the bloodstream. Moreover, the relatively low partial pressure of molecular oxygen in biological tissues may strongly limit singlet oxygen photogeneration, particularly under hypoxic conditions; experimental studies have shown that ^1^O_2_ generation decreases markedly as oxygen partial pressure falls. As a result, the relationship between photochemical damage and irradiance becomes nonlinear: increasing the power density does not necessarily enhance the effect and, under certain conditions, reduces the total generation of singlet oxygen [5,7,10,11,12,13]. Strategies for overcoming hypoxia include reducing the fluence rate, fractionation with intervals for reoxygenation, oxygen-generating and catalase-like nanomaterials, modulation of vascular perfusion, and the use of photosensitizers with an increased contribution of type I reactions [8,13,14,15,16,17,18,19,20,21,22]. However, the effectiveness of each strategy depends on the initial perfusion, tissue depth, oxygen consumption rate, photobleaching, and temporal dynamics of the vascular response; a universal regimen for all tumors is physiologically unlikely.

A third fundamental limitation is based on the lack of a directly measurable photodynamic dose. The nominal light dose does not take into account the local concentration of the photosensitizer and oxygen, and the pre-treatment drug concentration does not reflect its subsequent photobleaching, redistribution, and changes in quantum efficiency in the tissue microenvironment. Modern dosimetry therefore includes explicit approaches that measure light, the photosensitizer, and oxygen; implicit approaches, using fluorescence or photobleaching as a response surrogate; and direct approaches, targeting photochemical products [23,24,25]. None of these approaches have been universally accepted for all photosensitizers and clinical indications [5,6,7]. Image-based planning and Monte Carlo simulations allow for the calculation of the spatial distribution of irradiance and fluence, but require individual data on anatomy, optical properties, and the location of light sources [18]. Furthermore, measurement errors in each input parameter accumulate and can significantly impact the final assessment.

### 1.3. Why PDT Is a Good Field in Which to Exploit Artificial Intelligence

Inter-patient differences and high intertumoral heterogeneity make precision prognosis hard. The receptor profile, vascular density, stromal components, antioxidant capacity, activity of repair systems, hypoxic signatures, immune infiltration, and previous treatment can alter sensitivity to the same combination of photosensitizers and light.

Even in a single cancer tumor, the concentration of the photosensitizer, availability of oxygen, and viability of cells can be different on the millimeter scale. In clinical practice, such parameters are evaluated separately or completely ignored and are not included in the treatment plan. Due to this, optimization rests on average protocols and limits the number of indicators, but not on a quantitative model of a certain patient and the dynamics of the procedure [26,27,28,29].

PDT is a suitable method for AI because it is driven by many parameters that vary over time. Linear models lacking predefined nonlinear terms and interactions provide a limited description of the combined effects of photosensitizer concentration, fluence rate, oxygenation, perfusion, and photobleaching. Machine learning methods offer the potential to model more complex relationships but require stronger external validation.

Internal data have multimodal characteristics, including chemical structures and molecular descriptors, quantum-chemical parameters, absorption and fluorescence spectra, microscopic images, CT and MRI scans, optical spectroscopy data, genomic and transcriptomic profiles, clinical characteristics, and time-series sensor signals. It is important to gather these levels, but not to use a separate classifier for tables; they are more perspective in AI–PDT [14,15,16,17]. In the case of AI, the main benefit is the ability to analyze data that are different in scale and nature.

Molecular signs describe the potential ability of a compound to absorb light and form the triplet state. Characteristics of nanoformulation describe transport and release. Visualization data characterize the geometry, perfusion, and heterogeneity of the tissue. Spectroscopic signals provide information about the photosensitizer concentrations, photobleaching, and oxygen saturation.

In traditional analysis, this level can be reviewed independently. Due to this, multimodal links can always be lost. Multimodal models can combine them into a single feature space, albeit only with the correct time synchronization, standardization of units, and consideration of the uncertainty of each measurement.

In the first stage, AI can facilitate the search for photosensitizers by exploiting QSAR/QSPR models, molecular graph-theory-derived descriptors, including topological descriptors, graph neural networks, quantum-chemically informed predictors, and generative designs. Such models potentially allow for excluding molecules with non-beneficial spectra, low generation of active oxygen forms, higher dark toxicity, and difficult synthesis. At the formulation stage, the algorithm can predict the size of the particles, polydispersity, the ζ-potential, the efficiency of encapsulation and stability, and the release profile. In the current medicine stage, surrogate neuron models can facilitate the calculation of light propagation, and an optimization algorithm determines the number and position of LEDs, as well as power density and the fraction mode. After education begins, analysis of fluorescence, reflectance spectra, oxygenation, and images can be supported in real time. Finally, multimodal prognostics models can link the characteristics of patients, medical treatment, and the probability of a full response, local recidivism, toxicity, and immune response [14,15,16,17,18,19]. Another approach is to link physically informed machine learning. In such models, optimization is restricted by known laws of light transport, oxygen diffusion, photochemical kinetics, or pharmacokinetics. It reduces the feasible solution space and improves tolerability in small samples. A neural network can quickly approximate Monte Carlo calculations. However, its outcome has to be able to safely balance and behave correctly outside of the studied range. Similarly, the prognosis of molecular properties has to conclude model portability to new chemical frames, not only a middle error on a randomly selected sample. Due to this, the most realistic architecture is a hybrid system, where mechanistic equations set up physical boundaries of the system, and learning sets up parameters, which are rarely measured indirectly [30,31,32,33].

Not only do the discrimination properties of the model in the case of clinical use matter, but calibration, robustness to changes in equipment, and the ability to inform about uncertain conditions in the case of the model also matter. An algorithm that makes precise predictions in studying cohorts in research cohorts can be dangerous in transmission with another photosensitizer, light source, and visualization protocol. Consequently, AI has to be included in a quality control system, where processes such as data drift, changes in feature distributions, and patient compliance affect the model’s applicability domain. Such a setup makes PDT a unique example of personalized medicine, in which calculations can be personalized with the tight integration of the algorithm to the experimental design, dosimetry, and clinical context.

### 1.4. Purpose, Restrictions, and Scientific Questions of the Review

**Figure 1:**
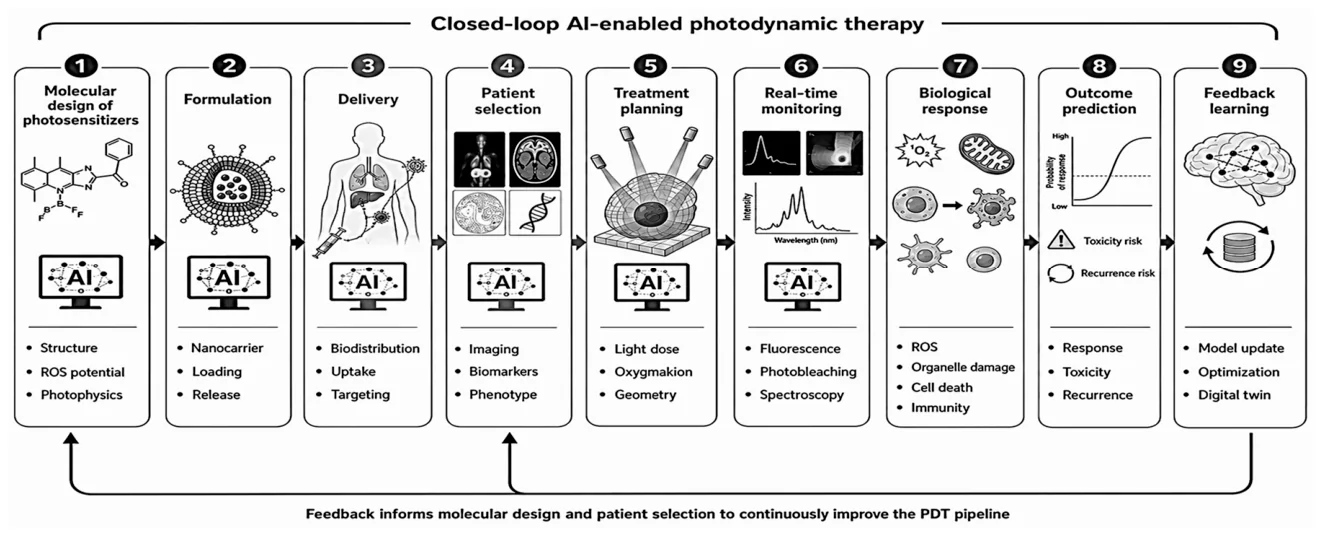
AI across the PDT pipeline.

The purpose of the present review is to analyze the ability of AI to transform the full cycle of PDT, from the molecular design of photosensitizers to intelligent delivery, the adaptive dosimetry of light, and personalized result calculation. The central concept is the closed AI–PDT cycle. Several recent reviews have addressed selected aspects of artificial intelligence in PDT, including AI-assisted photodynamic diagnosis and therapy, machine learning approaches in molecular photonics, and AI-supported optimization of photosensitizer-based nanopharmaceuticals. In contrast, the present review considers the complete AI–PDT workflow as an interconnected closed-loop framework, linking molecular photosensitizer design, nanoformulation and delivery, treatment planning, adaptive dosimetry, real-time monitoring, multimodal response assessment, and clinical outcome prediction. Particular emphasis is placed on the distinction between general AI applications and approaches specifically validated in PDT, as well as on data standardization, mechanistic constraints, external validation, and clinical readiness. First, the molecular structure determines the photophysical and pharmacological properties of the PDT agent. Formulation is dependent on photophysical and photochemical properties and has an influence on tissue distribution. Second, medicine distribution, oxygen, and light form the local photodynamic dose. Molecular damage determines cells, vessels, and immune reactions. Clinical results move back to the system as data to update the model. This analysis allows us to review AI not only as an isolated instrument but as a connection chain between photosensitizer chemistry, nanomedicine, the biophysics of light, and the molecular biology of response and clinical decision-making. The pipeline of using AI in PDT is described in Figure 1.

In the review, we consequently analyzed (i) experimentally supported stages of PDT, with well-checked algorithms, which remain conceptual; (ii) how certainly can we prognose spectral, photochemical, pharmacokinetic, and toxicological properties of new photosensitizers; (iii) can AI facilitate the performance of nanoformulation and address delivery; (iv) the compositions of optical, pharmacological, and physiological parameters to be used for personalized dosimetry; (v) what visual, molecular, and oxime biomarkers can be most informative to the physiological response; and (vi) what requirements must be provided for data, interpretation, regulation, external validation, and security before clinical implementation. Particular attention is focused on the distinction between computational proof-of-concept and technology that has undergone independent, prospective, and multicenter validation, as well as the need to maintain meaningful human control over the algorithm’s output [34,35,36].

## 2. Molecular and Clinical Basis of Photodynamic Therapy

Photodynamic therapy PDT is a simultaneous spatial and managed photochemical technology, in which the therapeutic effect develops in the photosensitizer, light, and molecular oxygen, which are simultaneously present. The simplicity of this triad is deceptive [37,38,39,40]. The resulting response forms in the system and contains many levels of organization, which include the electronic structure of the photosensitizer, its aggregation and pharmacokinetics, the optical properties of the tissue, local oxygenation, intracellular localization, antioxidant capacity, vessel reactions, and the immune context. Because of this, the same light dose can produce different levels of ROS production. This leads to different types of cell death and non-standardized results [41,42,43,44].

Modern reviews show that PDT must be examined not as a fixed protocol of illumination, but as dynamic purpose chains, where the next level depends on the previous and is modified by the reverse loop feedback [45,46,47]. For artificial intelligence, this ability has principal meaning. An informative model must integrate, not separate, the meaning of the variable and the totality of molecular, biophysical, and clinical variables.

For AI–PDT modeling, endpoints must be defined across molecular, cellular, vascular, and clinical levels because no single measurement adequately represents the complete photodynamic response. Due to this, an appropriate AI–PDT model must connect the levels and timeline of events. Predictors, measured during the procedure time and before lighting, and outcomes are not allowed to be connected without time measurement. Consequence and time positioning decrease information leakiness and make prognosis more acceptable not only for the classification of responses but also for choice-changeable parameters.

### 2.1. Photophysics and Photochemistry Processes

After absorbing a photon, the photosensitizer turns from the singlet ground state S_0_ to one of the excited singlet states, quickly relaxing to S_1_. There are a few competitive pathways after that: fluorescence, internal conversion with energy dissipation as heat, and intersystem conversion to the triplet state T_1_. T_1_ has a longer lifetime in comparison to S_1_, and allows interaction with molecules of the microenvironment. The probability of such a transition is determined by spin–orbit interactions, energetic disruption between the singlet and triplet states, molecular carcass rigidity, and the presence of heavy atoms and the central metal. The polarity and viscosity of the media also contribute. Modern design strategies are focused on increasing the triplet state and managing competition between the triplet state, energy transfer, and electron transfer [48,49]. It can have a characteristic influence at the molecular data level, which can be described by quantum descriptors and the development of QSPR and graph models.

In the classical Type II mechanism, the excited triplet photosensitizer transmits energy to molecular oxygen in the middle triplet state, with generation of a singlet oxygen ^1^O_2_. Because of its short lifetime and limited diffusion distance, ^1^O_2_ primarily damages biomolecules close to its site of generation. In the case of the Type I mechanism, the transfer of an electron or hydrogen atom occurs, forming the radical intermediates present. From radical intermediates, the next type of compounds is generated: a superoxide anion, hydrogen peroxide, and, in the presence of transition metals, the hydroxyl radical. In real biological systems, Type I and Type II rarely coexist as completely isolated routes. The feasibility and relative contribution of Type I and Type II photoreactions can also be mechanistically evaluated using quantum-chemical calculations, including analysis of excited-state energetics, electron-transfer pathways, and energy transfer to molecular oxygen. The relative contribution of these routes changes under different oxygen concentrations and donor and acceptor properties of the environment, aggregation, and local substrate concentration [5,48,49,50]. Hence, binary classification of photosensitizers as “Type I” and “Type II” is simplified. For efficacy prognosis, it is preferable to use the ROS profile. It takes into account the outcome of ^1^O_2_, the velocity of free radical generation, its dependence on _p_O_2_, and photochemical stability [51,52,53,54,55,56].

During irradiation, photosensitizer concentration, fluorescence, oxygen availability, and photobleaching dynamically change and therefore influence the effective photodynamic dose. Due to this, the light exposure dose in J is not a complete measure of the photodynamic dose. Modern dosimetry has a trend to take into account local light intensity, concentrations of the photosensitizer and oxygen, and the dynamics of photobleaching [5,57,58].

### 2.2. Molecular Properties of Photosensitizers as a Determinant of Efficiency

An ideal photosensitizer must combine intensive light absorption in the therapeutic window, a triplet state and higher ROS yield, low dark toxicity, chemical stability before activation, suitable solubility, and selective accumulation of the target. These requirements always conflict.

Photosensitizer performance is therefore determined by a balance between spectral properties, aggregation, solubility, and biological distribution [48,49]. For AI models, it is not enough to use only a two-dimensional structure: it is necessary to fix the solvent and pH, aggregation degree, concentration, excitation wavelength, and method of measurement.

The key quantitative parameters are maximum adsorption λ_max_, molar coefficient of extinction, quantum yield of fluorescence, triplet state and singlet oxygen, excited-state lifetime, logP/logD, _p_Ka, solubility, and photostability. However, even a high level of ΦΔ in vitro does not guarantee a pronounced therapeutic effect. Singlet oxygen diffuses over very small distances and is quickly quenched by biomolecules. Hence, photosensitizers with moderate ΦΔ and localized in the cell membrane may be more effective than those with ΦΔ distributed by diffusion. Likewise, positive charge allows interaction with negatively charged membranes and mitochondria, but can facilitate nonspecific interactions. Amphiphilicity improves membrane penetration. However, excessive lipophilicity impairs aqueous dispersibility.

Thus, molecular design is a multi-criterial task, where the optimum is determined not by maximization of one indicator, but by the compromise between photochemistry, pharmacology, and biological addressing [5,45,48].

Subcellular localization depends on molecular charge, lipophilicity, size, targeting ligands, and delivery strategy. Experiments with plasma membrane association showed that the delivery of ^1^O_2_ to the vulnerable membrane can simultaneously increase local damage and use limited oxygen more economically [55,56,57,58,59,60]. This result demonstrates that organelle localization can be independent of AI–PDT but cannot be measured as the common concentration of the photosensitizer in the cell.

### 2.3. Subcellular Targets and Forms of Cell Life

Photochemical reactions primarily damage lipids, proteins, and metabolites and, to a lesser extent, nucleic acids. Due to the short lifetime of the singlet oxygen, the damage occurs near the place of generation. The biological consequences depend strongly on subcellular localization, with mitochondrial, lysosomal, endoplasmic reticulum, and plasma membrane damage activating distinct death pathways. This type of cell death is determined not only by full ROS production, but also by the spatial and temporal distribution of the damage, the intensity of influence, and the ability of the cell to activate adaptive programs.

Depending on dose and localization, PDT may induce apoptosis, necrosis, autophagy-associated responses, ferroptosis, necroptosis, or mixed phenotypes [53,54,61,62]. These endpoints are relevant to AI models because simple viability measurements do not distinguish mechanistically different responses.

Most therapeutically valuable is not only the simple death of the cancer cells, but also immunogenic cell death (ICD). ICD is characterized by damage-associated signals, including calreticulin exposure and release of ATP and HMGB1, which promote dendritic cell activation and adaptive antitumor responses [10,50,51,52]. The ability to induce ICD is different between photosensitizers and depends on the dose, localization, and mode of irradiation. Porphyrinic photosensitizers demonstrate markers of ICD in vitro and anticancer activity in vivo [51]. Nanoformulation can increase these effects by combining photosensitizer, immunomodulator, and blocker control points [10,52]. It is important to include not only the percentage of viable cells, but also the DAMP, the phenotype of dendritic cells, and parameters of the lymphocyte infiltration for the future prognosis model.

### 2.4. The Cancer Microenvironment, Vessels, and Immune Components

The cancer microenvironment is an active modifier of PDT. Chronic hypoxia restricts Type II photochemistry before the beginning of irradiation. Acute consumption of oxygen during the procedure facilitates its deficiency. Uneven perfusion, pO_2_, extracellular matrix density, acidosis, and antioxidant capacity create spatially heterogeneous PDT responses. The balance between direct cellular and vascular effects also depends on photosensitizer distribution and the drug–light interval.

The immunological result is also two-sided. PDT-induced inflammation and ICD may enhance antitumor immunity, whereas hypoxia and immunosuppressive components of the tumor microenvironment may limit this response. Modern data confirm a combination of PDT and immunotherapy, but optimal order, dosage, and intervals remain context-dependent [10,52,63]. As a result, the immune response should be reviewed as a dynamic endpoint, dependent on the microenvironment, cell death characteristics, and the extent of vascular damage.

### 2.5. Clinical Protocol and the Concept of the Efficient Photodynamic Dose

The clinical protocol is set by choosing the photosensitizer, dose, input method, drug–light interval, wavelength, fluence, fluence rate, duration and fractionation of illumination, field geometry, and type of light guide. Wavelength, fluence, fluence rate, fractionation, and light-source geometry jointly determine the spatial and temporal photodynamic dose; therefore, identical nominal surface doses may produce different tissue-level effects [59].

**Figure 2:**
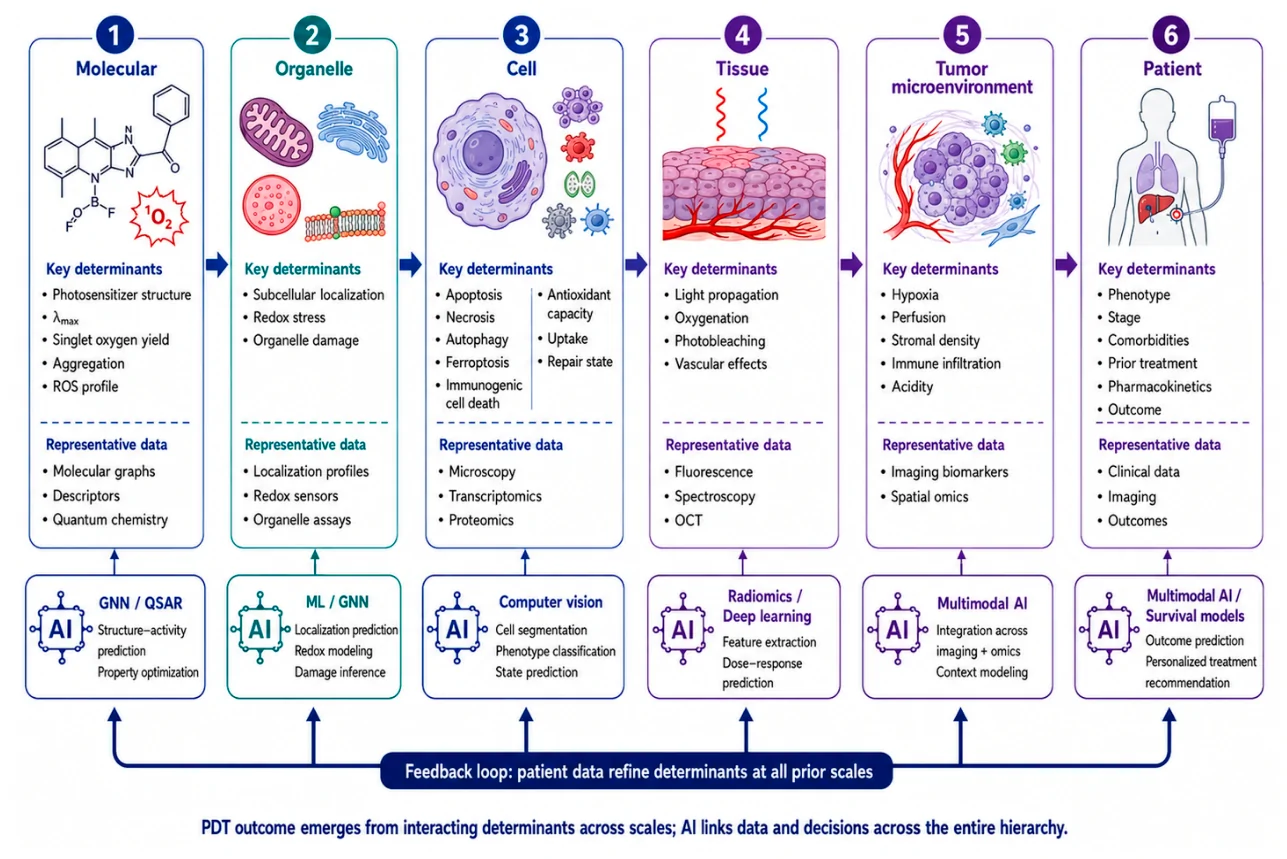
Multiscale determinants of PDT outcomes.

Personalized dosimetry is divided into explicit, implicit, and direct/biological approaches. Explicit dosimetry truly measures three main components—light, photosensitizer, and oxygen. It models ROS production. Implicit dosimetry uses photobleaching or other indirect signs. Biological approaches evaluate different changes in the tissue, vessels, and molecular markers. No single dosimetric approach is universal because optical and biological parameters vary spatially and dynamically during treatment. A modern review of dosimetry highlights the necessity of multichannel models for monitoring that are able to update evaluations of the dose in real time [6,57]. This is a direct entry point of physics-informed machine learning, where light transfer physical restrictions of oxygen consumption are combined with patient data. Management of patients’ data, using multiscale determinants, is presented in Figure 2.

Thus, the clinical effect of PDT appears from the nonlinear interaction of molecular structures, intracellular distributions, light, oxygen, and biological reactions. Neither the concentration of the photosensitizer nor fluence nor any biomarker describes this system separately. To use AI, we need a few levels of presentation: chemical photostage descriptors, pharmacokinetics and localization, three-dimensional light distribution, dynamics of oxygenation and photobleaching, cell death signs, vessel damage and immune activation, clinical outcomes, and toxicity. Thus, this setup allows for switching from correlating to prognosing to grounded adaptation protocols. Molecular biophysics determines the requirements for the algorithm, data type, and validation, which are reviewed in the next section.

## 3. Artificial Intelligence Technologies Relevant to Photodynamic Therapy

### 3.1. From Traditional Machine Learning to Deep and Generative Models

Artificial intelligence in PDT should be regarded not as a single algorithm, but as a set of computational approaches that are capable of solving tasks at various levels, ranging from the analysis of the molecular structure of the photosensitizer to the selection of irradiation regimens and the prediction of clinical response. The current literature describes the emergence of an integrated AI–PDT framework in which data from chemistry, photobiology, imaging, and clinical practice are combined into a sequence of decision-support models [14,15,16,64]. In this context, the choice of algorithm is determined not by the trendiness of the architecture, but by the type of data, sample size, nature of the target variable, and the need to interpret the result.

For the relatively small tabular datasets typical of preclinical PDT, interpretable regression and regularized models remain useful, whereas random forests, gradient boosting, XGBoost, and support vector machines can capture nonlinear interactions among photosensitizer concentration, fluence rate, oxygenation, and treatment response.

Deep learning architectures are particularly relevant when PDT data have spatial or temporal structures, including microscopy, OCT, fluorescence and hyperspectral images, and time-series measurements of photobleaching or oxygenation. The application of YOLOv3 to images of cells after PDT has demonstrated the ability to automatically distinguish and count live and dead cells, transforming subjective morphological assessment into reproducible quantitative analysis [65,66]. In ophthalmology, deep learning has been used for three-dimensional segmentation of fluid regions in OCT and for assessing responses to photodynamic therapy [67]. These examples illustrate that AI can optimize not only the treatment decision but also the method of measuring endpoints.

Transformers, attention mechanisms, and generative models may support molecular design and multimodal PDT analysis, including the processing of SMILES representations, images, treatment parameters, and longitudinal data. However, synthetic data should not automatically be considered equivalent to experimental data: they can reproduce latent biases (displacements) from the source dataset and generate examples that are chemically implausible or clinically unrepresentative. Therefore, generative models in FDT must be used in conjunction with physicochemical constraints, uncertainty assessment, and experimental validation.

### 3.2. Molecular Representations, Graph Networks, and Multi-Task Learning

Accurate computational representation of the molecule is critical for predicting the properties of photosensitizers. Classical QSAR/QSPR approaches employ molecular descriptors and fingerprints such as molecular weight, polar surface area, the number of hydrogen bond donors and acceptors, logP, topological indices, electronic parameters, and fragment-based features. While such representations are convenient for small datasets and allow for the use of standard algorithms, some structural information is lost during the encoding stage.

Graph neural networks encode atoms and bonds directly and can learn structural features associated with conjugation, aromaticity, charge, coordination centers, and substituent effects. HiGNN combines a full molecular graph representation with chemically meaningful BRICS fragments, thereby enhancing prediction accuracy and facilitating the identification of structural motifs that determine properties [68]. Recent research indicates that performance depends not only on network depth but also on the message aggregation method, the incorporation of geometry, and the representation of the 3D structure [69,70].

Multi-task learning holds particular promise for PDT; in this approach, a single model simultaneously predicts the absorption maximum, quantum yield of singlet oxygen, solubility, lipophilicity, dark toxicity, and phototoxicity. However, the multi-task approach can yield poorer results if measurements are obtained using incompatible solvents, different wavelengths, or biological systems of varying complexity. Consequently, the dataset must encode experimental conditions rather than just the compound’s structure.

Accordingly, measurements obtained under incompatible experimental conditions should not be pooled as if they represent identical endpoints.

The machine learning in question has already demonstrated the ability to link structural and electronic features to two-photon absorption [71]. For photosensitizers, a similar logic can be applied to analyze the factors determining red-shift, intersystem crossing, and ROS generation. Nevertheless, the model should not replace quantum chemistry. A hybrid scheme is the most robust approach: DFT or TD–DFT generate physically meaningful descriptors, machine learning accelerates screening, and experiments validate the predictions.

### 3.3. Supervised and Unsupervised Learning, Transfer Learning, and Active Learning

A major challenge in PDT is that many datasets are small and measurements are heterogeneous. Consequently, random splitting into training and test sets can create an illusion of high accuracy, particularly if closely related chemical analogs, images from the same patient, or technical replicates end up in different subsets. For molecules, scaffold splitting is preferable, whereas for images, splitting should be performed at the patient or experimental series level.

Unsupervised methods such as PCA, t-SNE, UMAP, and autoencoders can identify groups of photosensitizers or cellular response phenotypes, although the resulting clusters remain dependent on preprocessing and do not establish biological causality.

Transfer and self-supervised learning may reduce the amount of expert annotation required for PDT imaging, but their validity depends on sufficient similarity between the source and target domains. Since natural images, standard radiology, and fluorescence microscopy exhibit distinct statistical characteristics, it is essential to apply the domain shift check.

Active learning offers a particularly efficient strategy for costly photochemical experiments. Active learning and Bayesian optimization can prioritize the next photosensitizer or irradiation condition to test while reducing the number of costly experiments. In the context of PDT, this could involve selecting the next combination of concentration, fluence rate, and fractionation interval. However, the reward function must account not only for maximizing tumor cell death but also for preserving normal tissue, managing oxygen depletion, and adhering to technical constraints.

### 3.4. Physics-Informed AI and Digital Twins

Physics-informed models can constrain AI predictions using established descriptions of light transport, oxygen kinetics, photochemistry, and photobleaching, thereby reducing physically implausible extrapolation.

The practical value of physics-informed learning is evident in the assessment of tissue optical properties: a neural network augmented with physical relationships recovered absorption and scattering coefficients faster than traditional iterative procedures [72]. This is crucial for PDT planning, as identical light source parameters result in different spatial dose distributions in tissues with varying optical properties. Hybrid AI–Monte Carlo models can leverage accurate simulations to train a fast surrogate model suitable for interactive planning.

### 3.5. Explainability, Uncertainty, and Reliability Criteria

A clinically oriented AI system must provide not only a prediction but also the rationale behind the decision, the range of uncertainty, and the conditions under which the model is unreliable. Recent reviews emphasize that a visually compelling heatmap does not guarantee causal correctness: the explanation may be unstable, dependent on the architecture, and highlight a correlating yet biologically irrelevant background [73,74,75,76].

In PDT, explainability is particularly important due to the potential for hidden technical features. A model might recognize the microscope series, image brightness, plate type, or laboratory rather than the true biological response. In clinical data, institutional protocols, equipment types, and patient selection serve as similar sources of bias (displacement). That is why the interpretation should include sensitivity analysis, validation at an external center, counterfactual examples, and an assessment of whether the prediction changes in a biologically expected direction.

For chemical models, it is necessary to define the domain of applicability; for clinical models, the task is to identify patients who differ from the training cohort. A model’s refusal to provide an answer in an unusual case is preferable to a confident yet erroneous recommendation.

Reliability should not be assessed with a single type of metric. In medical imaging, it is essential to evaluate agreement with expert assessment and robustness against variations in imaging equipment. Prospective multicenter validation remains rare, although studies on AI-based skin spectroscopy demonstrate the feasibility of clinically validating devices with independent patients [77]. Similarly, AI-based analysis of OCT images following PDT shows potential for automating response assessment, yet its ability to move across different devices and populations requires further validation [67].

Consequently, the technological maturity of AI in PDT is determined not by algorithm complexity, but by a combination of sound data design, physical plausibility, interpretability, external validation, and experimental check-ups. Recent reviews on AI in photomedicine and oncology emphasize that real-world clinical implementation is limited precisely by these factors, rather than by a lack of computational architectures [78,79,80,81,82].

### 3.6. Multimodal Models, Data Standardization, and Computational Infrastructure

Real-world PDT is rarely described by a single data type. Molecular structure characterizes the potential properties of a photosensitizer, spectroscopy reflects its state in a specific environment, images show spatial distribution, and clinical parameters describe the patient and the treatment administered.

Multimodal models can combine molecular, spectral, imaging, and clinical information, but the integration strategy must preserve differences in data scale, timing, and measurement uncertainty.

Temporal synchronization of data is crucial for PDT. Photosensitizer concentration, oxygenation, photobleaching, and morphology change at various times following drug administration and the onset of irradiation. Consequently, simply pooling measurements without timestamps can lead to spurious associations. In an adaptive system, the prediction must be updated after each new spectral or visual observation.

The quality of multimodal AI is determined by data standardization. For chemical entities, SMILES, InChI, protonation state, solvent, and temperature are required; for light, we measure the wavelength, spectral width, fluence, and fluence rate; and for biological experiments, we evaluate the cell line, seeding density, oxygen content, and time points. Images must be accompanied by instrument parameters, scale, exposure, and the preprocessing algorithm. Without these metadata, the model will learn from laboratory-specific artifacts rather than from the PDT mechanism itself.

For photophysical endpoints, a minimum metadata standard should additionally include the solvent, pH, oxygen concentration, photosensitizer concentration, aggregation state, excitation wavelength, reference compound, and detection method. Measurements of λ_max_, fluorescence quantum yield, singlet-oxygen quantum yield, and related condition-sensitive endpoints should be pooled only when experimental conditions are sufficiently harmonized. Otherwise, the experimental conditions should be incorporated explicitly as model features or represented using hierarchical models so that laboratory-, solvent-, and protocol-related variation is separated from molecular structural effects. Biological endpoints should likewise retain information on the cell or tissue model, photosensitizer concentration, light dose, fluence rate, oxygenation, and measurement time point.

Computational workflows should report sufficient preprocessing, model parameters, software versions, and validation procedures to permit reproduction and assessment across institutions.

Thus, multimodal AI should be designed as a managed information system rather than an isolated neural network. Artificial intelligence methods and their potential roles in the photodynamic therapy workflow are described in Table A1. Its scientific value is determined by the transparency of data provenance, the alignment of temporal and spatial scales, and the ability to reproduce the entire process from the initial measurement to therapeutic recommendations. Furthermore, clinically significant endpoints and the minimum acceptable improvement over simple baseline models should be defined in advance, as a statistically accurate algorithm may not offer a practically useful advantage to the medical specialist or explorer.

## 4. Photochemical Characteristics and AI

### 4.1. Limitations of the Traditional Search for Photosensitizers

The development of a new photosensitizer is a multi-criteria problem in which improving one property is often accompanied by a deterioration in another. Increasing the π-conjugation length typically shifts the absorption band towards the red or near-infrared region. However, it can simultaneously enhance planarity, hydrophobicity, and molecular aggregation. The introduction of heavy atoms or a coordination center can increase the probability of intersystem crossing and triplet state population, but may also increase dark toxicity, accelerate non-radiative relaxation, or complicate pharmacokinetics. Therefore, the search for a photosensitizer cannot be reduced to maximizing a single parameter, such as the quantum yield of singlet oxygen. A viable candidate must combine absorption within the therapeutic window, sufficient photostability, controlled generation of reactive oxygen species, solubility, low dark toxicity, synthetic accessibility, and suitable subcellular localization [83,84].

The traditional “synthesis–characterization–biological testing” cycle explores only a small fraction of chemical space. Even within a single class, such as BODIPYs, cyanines, porphyrins, or AIE luminogens, thousands of combinations of substituents, linkers, charge, and central atoms are possible. Recent reviews of molecular strategies for PDT emphasize that photodynamic efficiency is determined by the combined action of electronic, steric, and environmental factors, rather than simply by a compound’s affiliation with a known chromophore framework. It is exactly where machine learning can replace brute-force screening with a prioritized selection of compounds exhibiting the most promising balance of properties [83,84].

### 4.2. Construction of Chemical and Photochemical Datasets

The reliability of an AI model is determined primarily by the quality of the dataset. For photosensitizers, a basic record should include a unique chemical structure, canonical SMILES and InChI, charge, protonation state, stereochemistry, solvent, concentration, temperature, the measurement method, and the standard used. PubChem provides a scalable infrastructure for chemical identifiers, structures, and biological annotations; however, values for photophysical parameters often must be extracted from primary articles. Automatic data aggregation that ignores the solvent and experimental protocol creates a false impression of a large dataset: the same molecule in water, an organic solvent, a protein-rich medium, or an aggregated state actually represents different photochemical entities [33,34,35,83,84].

Standardization of the quantum yield of singlet oxygen, ΦΔ, is particularly critical. Its values depend on the detection method, excitation wavelength, oxygen concentration, reference compound, and the photosensitizer’s ability to aggregate. Therefore, a useful unit for machine learning is not only “structure–property” but the “structure–conditions–property” triad. In an experimentally validated study on ΦΔ prediction, models that accounted for chemical descriptors and measurement conditions enabled virtual screening of Type II photosensitizers. A similar approach is required for λ_max_, fluorescence yield, triplet lifetime, and phototoxicity [83,84,85].

Accordingly, minimum metadata standards should be defined for condition-sensitive photophysical endpoints. For absorption wavelength, fluorescence quantum yield, and singlet-oxygen quantum yield, datasets should include the solvent, pH, oxygen concentration, photosensitizer concentration, aggregation state, excitation wavelength, reference substance, and detection method as a minimum. Measurements obtained under sufficiently comparable experimental conditions may be merged directly. In contrast, data generated under substantially different solvents, oxygenation states, aggregation conditions, or analytical protocols should either be modeled separately or incorporated through hierarchical models that distinguish molecular structural effects from laboratory- and protocol-specific variation.

**Figure 3:**
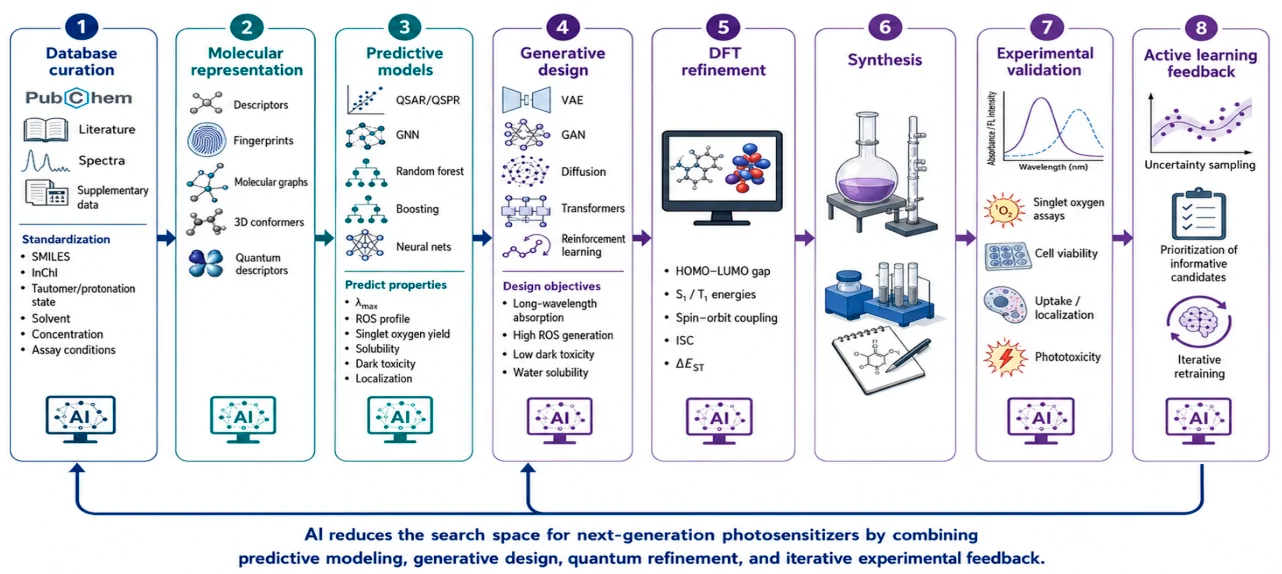
AI-guided photosensitizer discovery workflow.

Curation should include removing duplicates, verifying units, normalizing solvent names, controlling outliers, and flagging uncertainties. Failed compounds and low property values are just as important as successful results: the absence of negative data leads to a bias in the model toward published “winners”. A promising solution is the creation of an open registry of photosensitizers with raw spectra and metadata, where each numerical value is accompanied by a confidence interval and a link to the experimental protocol [35,83,85]. The usage of AI in PDT agent discovery is explained in Figure 3.

### 4.3. QSAR/QSPR Models and the Choice of Molecular Representation

In the QSPR approach, a molecule is transformed into a set of computable features: topological indices, fragment counts, molecular fingerprints, parameters of polarizability, charges, geometry, HOMO–LUMO gap, and the energy characteristics of excited states. For small datasets, interpretable models—multiple linear regression, support vector regression, random forest, and gradient boosting—often outperform deep neural networks because they require fewer examples and allow assessment of feature contributions. A QSPR model for heavy-atom-free BODIPY showed that singlet oxygen generation can be linked to combinations of structural and electronic descriptors, rather than solely to the presence of a halogen. A later study on BODIPY predicted the ratio of fluorescence to singlet oxygen yields and identified informative descriptors related to electronic structure and heteroatom distribution [83,84,86,87].

The choice of representation depends on the specific task. Fingerprints are effective for finding local structural analogies but do not adequately encode solvent effects and conformational dynamics. Graph neural networks learn representations directly from atoms and bonds; however, with a small number of photosensitizers, they may memorize scaffolds instead of identifying transferable patterns. Three-dimensional and quantum-chemical features better reflect intersystem crossing and charge transfer but increase computational cost. A reasonable compromise is a hybrid representation that combines RDKit descriptors, molecular fingerprints, calculated electronic parameters, and experimental environment features [35,86,87].

Models should be validated using more than just random splitting. With random splitting, close analogs of a single scaffold may end up in both the training and test sets simultaneously, inflating the accuracy. Scaffold splitting and temporal splitting better mimic the search for a new chemical family. Additionally, an analysis of the applicability domain is necessary: the model should indicate when a new structure differs significantly from the training examples and the prediction has high uncertainty [35,86,87].

### 4.4. Predicting Spectral and Photochemical Characteristics

The spectral range determines the depth of effective excitation and the compatibility of the photosensitizer with the light source. Machine learning already enables accurate prediction of the absorption and emission wavelengths of organic dyes, using experimental data instead of computationally expensive calculations for each molecule. For PDT, this is not sufficient: it simultaneously requires a T1 energy above the threshold for energy transfer to triplet oxygen, efficient intersystem crossing, and limited internal conversion to heat. Therefore, the objective function must be multidimensional [83,84,88].

Modern models directly predict ΦΔ. For pteridines and fluoroquinolones, a comparison of linear and nonlinear methods demonstrated high accuracy following the genetic selection of descriptors; interpretation using SHAP and ALE revealed features associated with the length of conjugated bonds and polarizability. For transition metal complexes, combining DFT and machine learning makes it possible to account for the electronic configuration of the metal, the ligand environment, and spin–orbit coupling. This approach is particularly important for Zn(II), Ru(II), and Ir(III) porphyrinates, as well as other coordination systems, where simple organic fingerprints do not fully describe the photostates [85,86,87,88,89,90].

Recent experimental studies of Ru(II) coordination complexes further demonstrate that photosensitizer optimization should extend beyond isolated spectral descriptors. A biquinoline-containing Ru(II) complex combined Type I/II photochemistry with oxygen-independent phototoxicity and immunogenic cell death, ultimately promoting antigen-presenting cell activation and CD8^+^ T-cell-mediated antitumor responses. These results indicate that hypoxia tolerance, dark toxicity, immune activation, and in vivo efficacy should be incorporated as coupled optimization endpoints in future AI-guided photosensitizer design [90].

It is essential to distinguish between predictions of potential photochemical capability and therapeutic efficacy. A high ΦΔ measured in a deuterated solvent does not guarantee high ^1^O_2_ generation in the cell due to aggregation, protein quenching, oxygen deficiency, and competition from Type I processes. Therefore, the next step should be multiscale models that link molecular properties to cellular accumulation, organelle localization, and biological response [83,84,85,86,87,88,89,90].

### 4.5. Integration of Quantum Chemistry and Machine Learning

TD–DFT, multi-configuration methods, and spin–orbit coupling calculations provide mechanistically meaningful information but do not scale well to hundreds of thousands of structures. ML surrogates allow for the preliminary prediction of S_1_, T_1_, ΔEST, dipole moment, and spectral peaks, reserving high-precision calculations only for a small number of candidates. A self-improving photosensitizer search system, combining first-principles simulations and Bayesian optimization, has shown that actively selecting the next batch of molecules is more efficient than uniform computational screening [85,88,90,91].

An important advantage of the hybrid approach is the ability to impose physical constraints. A candidate with a predicted high ΦΔ can be excluded if the calculated triplet energy is insufficient for oxygen sensitization or if the excited state is localized on a fragment prone to rapid photodecomposition. Conversely, a deviation of the ML prediction from DFT may signal an unusual mechanism worthy of experimental investigation. Therefore, quantum chemistry and AI should be viewed as complementary, not competing, levels of validation [85,90,91].

### 4.6. Generative and Multi-Criteria Design

Generative models are capable of creating structures beyond the scope of existing libraries. Variational autoencoders, transformers, graph-based generators, and diffusion models generate molecules within specified constraints, but without chemical filters, they often propose unstable or synthetically inaccessible compounds. For PDT, a scaffold-constrained design is preferred: a proven chromophore is retained, while the model varies substituents, donor–acceptor architectures, charge, and targeting fragments [83,84,88,91].

Machine learning-based designs of cyanine photosensitizers have demonstrated the ability to jointly predict ΦΔ and fluorescence yield, followed by a two-stage virtual screening of thousands of derivatives. A closed-loop hybrid system for Type I photosensitizers integrated candidate generation, computational evaluation, and experimental feedback. These studies mark a transition from retrospective QSPR to prospective design, in which the algorithm proposes molecules prior to their synthesis [85,91,92,93].

The objective function should form a Pareto frontier rather than a single ranking. One candidate may be optimal for fluorescence-guided PDT, another for a hypoxic tumor dominated by the Type I mechanism, and a third for antimicrobial applications. In multi-criteria optimization, it is advisable to include λ_max_, ΦΔ, or radical ROS yield, the extinction coefficient, solubility, logD, _p_Ka, the probability of aggregation, photostability, dark toxicity, synthetic complexity, and subcellular localization prediction [83,84,85,92,93].

### 4.7. Active Learning and the Experimental Closed-Loop Cycle

Active learning is particularly useful when experimental data collection is costly. The model selects not simply the compounds with the highest predicted values, but examples that are both promising and reduce uncertainty. A unified active learning framework with a graph network and adaptive acquisition functions has proven effective for discovering photosensitizing molecules and predicting S_1_/T_1_ energies. Although some such platforms are developed for the field of photovoltaics, their mathematical framework can be adapted to medical photosensitizers by replacing the objective functions [91,92,93,94].

The practical workflow includes automated data extraction, library generation, initial ML filtering, quantum-chemical calculation, assessment of synthetic feasibility, synthesis, spectroscopy, ROS measurement, cell screening, and model updating. Automation does not replace expert oversight: a chemist must verify reaction feasibility, a photobiologist must verify the accuracy of measurements, and a data specialist must ensure there is no data leakage or overfitting [35,91,92,93,94].

### 4.8. The Relationship Between Molecular Design and Biological Selectivity

The next generation of models must predict not only photochemistry but also the behavior of the photosensitizer within a biological system. A cationic charge can enhance binding to mitochondria and bacterial membranes, but it can also increase nonspecific cytotoxicity. Lipophilicity facilitates membrane penetration but promotes aggregation and binding to plasma proteins. A targeting ligand may improve uptake by cells with a specific receptor but alter the spectrum, size, and pharmacokinetics of the conjugate [83,84,92,93].

Training requires multimodal data: chemical structure, spectroscopy, cell images, concentration–time curves, transcriptomic response, and microenvironment parameters. Predictions of phototoxicity should be distinguished from dark toxicity, and the cell line, oxygen status, light dose, and time point should be included as explicit features. Only such a model will be able to distinguish between a “good ROS generator” and an “effective therapeutic agent” [35,85,92,93,94].

The distinction between photochemical performance and therapeutic performance is illustrated by the β-carboline/furylmalononitrile hybrid 10f. Its singlet-oxygen quantum yield increased approximately threefold as the pH decreased from 7.4 to 4.5, while the compound also demonstrated hypoxia-tolerant phototoxicity, mitochondrial damage, apoptosis/autophagy-associated responses, low acute toxicity, and an in vivo tumor-inhibition rate of 80.5%. Such multidimensional experimental profiles provide more clinically meaningful training labels for AI models than λ_max_ or ΦΔ alone [94].

### 4.9. Limitations and Evidence Requirements

The main obstacles are small datasets, inconsistent protocols, publication bias, a lack of external validation, and limited access to raw data. A high R^2^ in a randomly split sample does not prove the ability to identify a new scaffold. Independent test series, blinded prospective synthesis, and the publication of negative results are required. It is advisable to compare AI not only with other algorithms but also with basic chemical rules, similarity search, and standard DFT screening [35,85,86,87,88,89,90,91,92,93,94].

For clinically oriented designs, ADME, genotoxicity, photoproducts, metabolites, and the likelihood of long-term cutaneous photosensitivity should be additionally evaluated. Models of fluorescent quantum yields and aggregate states, web tools for BODIPY spectral properties, and general QSPR platforms for optical materials expand the technical foundation but require domain-specific adaptation to PDT. The most convincing chain of evidence is considered to be “interpretable prediction → synthesis → independent spectroscopy → cellular and in vivo validation” [85,86,87,88,89,90,91,92,93,94,95,96,97,98].

Thus, AI transforms the development of photosensitizers not by mechanically replacing the chemist, but through the rational navigation of the chemical space. Its main value lies in integrating structural, quantum-chemical, photochemical, and biological data into a multi-criteria decision-making system. With proper standardization and prospective validation, this approach can reduce the number of unpromising syntheses and accelerate the transition from a molecular hypothesis to a validated PDT agent [35,83,84,85,86,87,88,89,90,91,92,93,94,95,96,97,98].

Finally, the development of a photosensitizer must take into account the anticipated clinical scenario as early as the in silico stage. For superficial dermatological PDT, different spectral and pharmacokinetic trade-offs are acceptable compared with those in interstitial treatment of deep tumors. For antimicrobial PDT, cationicity, rapid binding to the microbial envelope, and minimal risk of resistance development are important; for oncology, selectivity, distribution within the tumor, and the immunogenicity of the damage are key. Context-dependent objective functions will shift the focus from an abstract search for the “best photosensitizer” to the design of molecules tailored for specific biological and clinical applications [83,84,92,93].

It is equally important to distinguish between a correlational interpretation and a causal molecular mechanism. High descriptor importance in SHAP analysis indicates its contribution to the prediction but does not prove that altering this feature will directly improve photosensitization. For example, the number of heavy atoms may correlate with ΦΔ in the training set, although the actual result depends on substituent position, orbital symmetry, state energies, and competing internal conversion. Therefore, interpretable ML results must be validated by targeted DFT calculations, transition-state spectroscopy, and the synthesis of controlled series of analogs. Such validation transforms the model from a statistical filter into a tool for constructing chemical hypotheses [86,87,89,90,91].

Another fundamental challenge is the transferability of models across chemical classes. Patterns identified for BODIPY do not necessarily apply to porphyrins, phenothiazines, cyanines, or metal complexes, since the mechanisms of intersystem crossing, aggregation, and intramolecular charge transfer differ. A universal model must either be trained on a sufficiently broad chemical space or employ a hierarchical architecture, where the general level encodes fundamental photophysical relationships, while specialized submodels account for scaffold-specific features. Transfer learning is a useful approach: the model is first trained on large datasets of spectral and quantum-chemical data, and then fine-tuned on a small but high-quality set of medical photosensitizers. However, the transfer will be accurate only if domain shift is controlled and uncertainty is explicitly assessed. Pre-training can leverage large photophysical datasets and hybrid ensemble models, cross-domain collections of photochemical properties, integrated chemical–biological representations from PubChem, and specialized models for predicting emission and quantum yields [88,95,97,98,99,100,101].

The practical value of a model increases if, alongside point predictions, it provides confidence intervals, nearest chemical analogs, and an explanation of risk factors. For a candidate outside the algorithm’s applicability domain, the algorithm should recommend an additional calculation or experiment rather than providing a definitive number. This approach aligns with the role of AI as a decision-support system, reducing the likelihood of costly synthesis based on an uncalibrated prediction [86,87,88,91,94,97,98,101].

Information about machine learning models for predicting photosensitizer properties, published examples, and limitations are gathered in Table A2.

## 5. AI-Optimized Delivery of Photosensitizers

### 5.1. The Challenges of Delivering Photosensitizers

The therapeutic activity of a photosensitizer is determined not only by its spectral and photochemical characteristics, but also by the concentration, physical state, and subcellular compartment in which it reaches the target. Many porphyrins, phthalocyanines, chlorins, BODIPY derivatives, and cyanines are characterized by pronounced hydrophobicity, a tendency towards π–π aggregation, and nonspecific binding to proteins. In the aggregated state, the proportion of photoactive monomers decreases, absorption and fluorescence spectra change, non-radiative relaxation is enhanced, and oxygen availability is reduced. Delivery systems therefore need to increase solubility, prevent premature aggregation, and preserve the sensitizer’s ability to generate reactive oxygen species (ROS) after release. Nanocarriers make it possible to combine these requirements with controlled pharmacokinetics, protection of the molecule from degradation, and increased accumulation in pathological tissue [102,103,104,105].

However, the challenge of delivery in PDT is fundamentally more complex than simply improving bioavailability. The carrier must ensure the coordination of at least four temporal processes: circulation and distribution of the drug, release of the photosensitizer, achievement of the maximum target/normal tissue ratio, and light delivery. In addition, endocytosis, endosomal escape, intracellular transport, and organelle targeting must be taken into account. If the photosensitizer remains firmly bound to the matrix, the ROS it generates may mainly damage the carrier; if it is released too rapidly, the advantage of targeted delivery is lost. Thus, the optimal formulation is a multi-criteria task in which size, charge, morphology, composition, stability, release profile, and photochemical activity must be optimized together [102,103,106,107].

### 5.2. Diversity of Nanocarrier Platforms

For PDT, liposomes and lipid nanoparticles, polymer micelles, nanogels, dendrimers, protein particles, mesoporous silica, metallic nanostructures, metal–organic and covalent organic frameworks (metal–organic frameworks, MOF; covalent organic frameworks, COF), exosomes, membrane-coated particles, and ligand–photosensitizer conjugates are used. Lipid systems are particularly suitable for encapsulating hydrophobic sensitizers and allow fine regulation of membrane fluidity, PEG coating, and ligand density. Polymer carriers provide a broader range of degradation rates and controlled release, while porous inorganic platforms have a high loading capacity and can combine PDT with imaging, photothermal therapy, or catalytic oxygen generation [103,104,105,106,107,108].

The choice of carrier should depend on the clinical scenario. For topical dermatological PDT, local retention and penetration through the stratum corneum are important; for intravenous oncological therapy, stability in blood, limited uptake by the reticuloendothelial system, and penetration into the tumor are crucial; for antimicrobial PDT, rapid binding to the microbial envelope and minimal toxicity to host tissues are essential. Subcellular targeting additionally requires control over the presentation of mitochondrial cations, lysosomotropic groups, or peptides. Modern polymer nanocarriers can direct the photosensitizer to mitochondria, lysosomes, or the endoplasmic reticulum, but increased specificity is often accompanied by greater synthetic complexity and product variability [104,108,109].

### 5.3. Machine Learning for Formulation and Manufacturing Optimization

**Figure 4:**
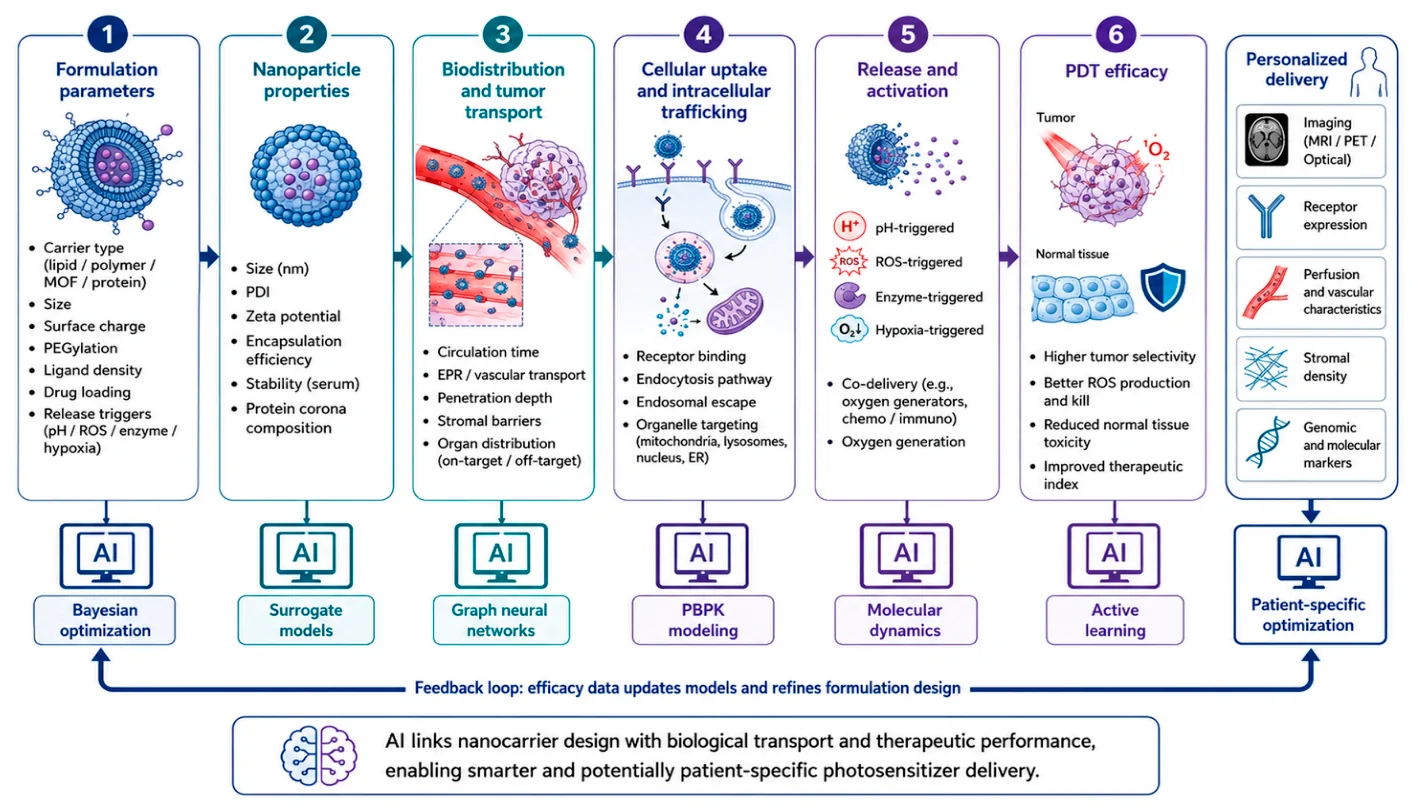
AI-guided nanocarrier optimization for PDT.

Traditional nanofomulation development is based on sequential variation of one or more factors and quickly becomes inefficient when dozens of components and process parameters are involved. Machine learning makes it possible to approximate the nonlinear mapping from composition and process to critical quality attributes. Input variables include the type and proportion of lipids or polymers, the photosensitizer/carrier ratio, phase concentrations, pH, temperature, mixing speed, flow rate in a microfluidic device, sonication time, and purification conditions (Figure 4). Output parameters are size, polydispersity index (PDI), ζ-potential, encapsulation efficiency, drug loading, stability, and release rate [110,111,112,113].

A telling example is the analysis of more than 1300 liposomes produced by a microfluidic method, in which XGBoost models predicted particle formation, size, and manufacturing parameters, while an interpretable analysis revealed the contribution of the chemical nature of the lipids and the mixing regime. In another study, ensemble learning was used to predict liposome size and PDI depending on composition, extrusion temperature, and processing time. These studies are important for PDT because photosensitizers often alter membrane packing, surface charge, and liposome stability; a model trained only on “empty” particles will not necessarily be transferable to a photoactive formulation [110,112,113].

For practical optimization, the most useful approach is a combination of design of experiments (DoE), the Quality by Design (QbD) concept, and machine learning. Experimental design provides controlled coverage of the factor space, the algorithm detects nonlinear relationships, and Bayesian optimization proposes the next series of experiments with the greatest expected improvement or information value. Such an active learning cycle is especially justified when the synthesis, spectral characterization, and biological validation of a single formulation are costly. Unlike an exhaustive grid search, it directs resources towards the Pareto-optimal region, where high encapsulation, the required size, a low PDI, and retention of photodynamic activity are achieved simultaneously [102,111,114].

### 5.4. Predicting Nano–Bio Interactions

After contact with biological fluids, the surface of a nanoparticle is rapidly coated with a protein corona, which forms its actual biological identity. The composition of the corona depends on size, curvature, charge, hydrophobicity, surface chemistry, incubation time, and plasma composition. It can mask targeting ligands, alter cellular recognition, activate complement, and redirect particles to the liver or spleen. For this reason, predicting only the initial physicochemical characteristics is insufficient: delivering a photosensitizer requires models that link engineered surface properties with the dynamic corona, cellular uptake, immune interactions, and pharmacokinetics [115,116,117,118].

Machine learning is already being used to predict the functional composition of the corona and the cellular recognition of nanomaterials. Models that combine nanoparticle features and protein properties make it possible to predict the enrichment of specific protein classes, and for DNA nanostructures, it has been shown that design can be linked to selective protein adsorption. For PDT, this is of direct importance: the binding to albumin, lipoproteins, and complement components can alter the delivery of porphyrin compounds, sensitizer availability, and the inflammatory response after irradiation. At the same time, transfer of models between different plasma types, diseases, and analytical platforms remains limited [115,116,117,118].

### 5.5. AI-Guided Targeting and Intracellular Delivery

Active targeting is based on the use of antibodies, peptides, aptamers, folate, transferrin, carbohydrates, and ligands to integrins or other receptors. However, the presence of a ligand does not guarantee improved delivery: the outcome depends on receptor density, the rate of its internalization, epitope accessibility, the protein corona, and the number of ligands on the particle. AI can integrate data on receptor expression, spatial transcriptomics, imaging, and carrier characteristics to choose not universal but context-dependent targeting. A similar approach applies to organelle targeting, where the model must take into account charge, lipophilicity, linker length, and release kinetics [108,109,119,120].

A promising example of method transfer is the AI-assisted design of ionizable lipids. Deep AGILE models and generative transformer platforms link the molecular structure of a lipid with delivery efficiency to specific cells, and AI screening of tens of millions of candidates has made it possible to obtain new lipids with efficiency comparable to or higher than that of control formulations. Although these systems were developed mainly for nucleic acids, their logic is applicable to PDT: the structure of an ionizable or helper lipid can be optimized for loading a specific photosensitizer, endosomal escape, and accumulation in a defined cell population [119,120,121,122].

### 5.6. Stimulus-Responsive Release and Modeling of the Microenvironment

Stimuli-responsive systems use an acidic environment, ROS, hypoxia, proteases, redox potential, temperature, ultrasound, a magnetic field, or light as a trigger. In PDT, a positive feedback loop is particularly attractive, in which the first doses of ROS break down a sensitive linker and release additional photosensitizer. However, excessively rapid degradation can cause local oxygen depletion, while a reaction threshold that is too high can render the system inactive. Machine learning models can predict release kinetics on the basis of composition, linker structure, pH, temperature, and irradiation parameters, while physics-informed approaches can constrain the prediction by the laws of diffusion and chemical kinetics [103,106,111,123].

A representative PDT-specific example is a Ce6-loaded hollow Prussian blue platform decorated with Au–Pt nanozymes. The nanozyme catalyzed the decomposition of tumor-associated H_2_O_2_ to generate O_2_, alleviated hypoxia, reduced HIF-1α expression, enhanced PDT efficacy, and suppressed pulmonary metastasis. For AI-guided formulation optimization, oxygen-generation kinetics, hypoxia imaging, HIF-1α modulation, metastatic burden, and conventional nanoparticle properties should therefore be modeled jointly rather than as isolated endpoints [123].

For a tumor, it is necessary to model not only the carrier but also the environment: vascular permeability, interstitial pressure, extracellular matrix density, oxygen concentration, and pH gradients. A multiscale framework can combine membrane molecular dynamics, a PBPK model of systemic distribution, CFD or a reaction–diffusion description of transport in tissue, and an ML surrogate model for rapid parameter exploration. Such hybrid approaches are preferable to a fully “black box” model, as they make it possible to check physical plausibility and carry out extrapolation more cautiously [102,117,120,123].

### 5.7. Personalization, Translation, and Data Requirements

Personalized delivery of photosensitizers must take into account the type and volume of the tumor, perfusion, vascular permeability, the proportion of stroma, receptor profile, liver and kidney function, immune phenotype, and prior treatment. Radiomic features may serve as non-invasive surrogate indicators of vascularization and heterogeneity, while omics data may point to a suitable ligand–receptor axis. Ideally, a decision-support system should compare several formulations and predict, for a specific patient, tumor accumulation, the risk of systemic photosensitivity, and the optimal drug–light interval [102,114,117,120].

The main limitation remains the gap between accurate prediction of size or encapsulation and real therapeutic efficacy. Datasets are often small, combine incompatible methodologies, and lack negative results. The same terms may denote different measurement protocols, and batch effects tie the prediction to a specific laboratory rather than to the causal properties of the formulation. AI-ready data should include the full composition, properties of the starting materials, process parameters, measurement methods, uncertainty, the photosensitizer, its spectral state, light parameters, the biological model, and time points [110,111,114,123].

Validation should be carried out sequentially: re-preparation of particles, blind verification of critical quality attributes, assessment of preservation of photochemical properties, testing in several cell models, in vivo pharmacokinetic and biodistribution studies, and then external validation in an independent laboratory. Data splitting must be achieved by formulation and batch, and when predicting new materials, by chemical scaffolds; otherwise, close analogs end up in both the training and test sets at the same time. The model should report its applicability domain and uncertainty, not just a point prediction [102,110,114,117].

Thus, AI is capable of transforming photosensitizer delivery from empirical carrier selection into controlled multiscale optimization. The greatest value lies not in autonomous algorithms, but in closed loops that combine standardized data, physical models, automated synthesis, and experimental feedback. For PDT, the ultimate objective function should not be the ideal nanoparticle in itself, but a reproducible photodynamic dose in the right tissue with minimal damage to normal structures. It is precisely the shift from formulation characteristics to the causal chain “delivery → localization → ROS → biological response” that will determine the clinical relevance of AI-guided nanomedicine [102,103,104,105,110,117,123].

Particular attention is required for endosomal escape, as a large fraction of nanoparticles after receptor-mediated endocytosis is directed to late endosomes and lysosomes. For photosensitizers, this can be both an advantage and a limitation. Lysosomal PDT can cause membrane permeabilization, release of cathepsins, and subsequent mitochondrial dysfunction; however, a photosensitizer intended for another organelle must leave the endosome before carrier degradation. Ionized lipids, proton-sponge polymers, fusogenic peptides, and photochemical internalization provide different escape mechanisms. An AI model can link material structure, apparent _p_Ka, buffer capacity, size, shape, and lipid composition with the probability of endosomal release; however, the target variable should be measured directly, rather than replaced by general transfection or cytotoxic activity [108,119,120,121,122,123].

Photochemical compatibility of the carrier should also be included among the critical quality indicators. Some matrices quench the excited state of the photosensitizer, compete for ROS, or alter the balance between Type I and Type II reactions. Metal and semiconductor components may enhance absorption or intersystem crossing, but catalyze dark redox processes at the same time. Therefore, formulation characterization should include absorption and fluorescence spectra, excited-state lifetimes, ΦΔ or validated ROS markers, photobleaching, and post-storage stability. For machine learning, these parameters must be linked to the sensitizer’s state inside the particle and after release; otherwise, optimization of drug loading may select a system with high loading but low availability of the photoactive monomer [103,104,105,106,107,108].

Scaling up creates an additional domain shift. A formulation optimized in a microliter device may change in size, morphology, and component distribution during the transition to continuous manufacturing. The outcome is affected by mixer geometry, Reynolds number, flow rate, order of addition, raw material batches, and sterilization methods. Models should be trained on process-aware data and separate molecular composition from equipment parameters. A useful strategy is transfer learning: a base model is trained on a large inter-laboratory dataset and then calibrated on a small amount of data from a specific production line. Prospective manufacturing runs and drift monitoring are essential, since gradual changes in raw materials or sensors can silently reduce prediction accuracy for the operator [110,111,112,113,114].

The clinical readiness of AI-optimized delivery should be assessed not by the model’s maximum accuracy, but by its ability to reproducibly improve decisions compared with rational experimental design. The minimum level of evidence includes an independent test set, blind synthesis of the proposed formulations, comparison with a clinically or experimentally established carrier, and demonstration of an improved therapeutic index in vivo. A higher level requires evaluation of scalability, stability, sterility, immunogenicity, and compatibility with the specific light source. Only then can the algorithm be used as part of a regulated manufacturing or clinical process. This approach prevents biomedical efficacy from being mistaken for statistical success and aligns AI-driven development with Quality by Design principles [102,110,114,117,123]. More information about the AI-oriented development of photosensitizer delivery systems and direct and transferable approaches is in Table A3.

Finally, for photosensitizers, the relationship between the carrier and the light protocol must be evaluated. Particle size and composition can alter the local absorption coefficient, scattering, the effective excitation depth, and photobleaching kinetics. Therefore, the same formulation is not optimal for all wavelengths, power densities, and fractionation regimens. Future models should jointly optimize the carrier composition, the drug–light interval, and irradiation parameters, using photochemistry and tissue optics as physical constraints. Such co-optimization links the delivery section with personalized dosimetry and turns the formulation into part of a single therapeutic protocol rather than an isolated pharmaceutical product [102,103,104,105,106,110,123].

## 6. Piece Intelligence for Light Delivery, Dosimetry, and Optimization of Photodynamic Therapy Protocols

### 6.1. Limits of the Nominal Light Dose

In clinical practice, light exposure during PDT is often described in terms of power density (or irradiance) and integral energy exposure (or fluence). These measures are necessary for standardization, but are not equivalent to a biologically effective photodynamic dose on their own. The same amount of energy delivered to the surface can create fundamentally different distributions of light within tissue because of differences in absorption and reduced scattering coefficients, tumor geometry, blood content, the extent of necrosis, and the spatial distribution of the photosensitizer. Additional nonlinearity is introduced by dynamic oxygen consumption, photobleaching, and PDT-induced changes in perfusion. For this reason, modern dosimetry should regard light not as a single therapeutic agent, but as one component of a time-varying photochemical system [6,57].

For superficial lesions, error can already arise at the stage of illuminating an uneven anatomical surface: the distance and angle of incidence alter local irradiance, while shadows, curvature, and uneven reflectivity create zones of under-irradiation and overheating. For deeply seated tumors, the problem is even more complex, since interstitial light guides create overlapping three-dimensional fields that are sensitive to millimeter-scale positioning errors. Accordingly, personalizing the protocol requires joint consideration of anatomy, optical properties, source characteristics, permissible doses for critical structures, and the temporal kinetics of the photochemical reaction [18,124,125,126,127].

### 6.2. Modeling Light Transport and Rapid Surrogate Models

Photon propagation in biological tissues is modeled using Monte Carlo methods, the diffusion approximation, and the finite element method. The Monte Carlo method remains the most versatile approach for complex geometry and heterogeneous media, as it directly reproduces the probabilistic acts of scattering and absorption. Its limitation is high computational costs, especially when the plan must be recalculated repeatedly if the positions of light guides, optical parameters, or source power change. Diffusion and finite element models are usually faster but require the relevant physical assumptions to be met and may lose accuracy near sources, boundaries, and low-scattering regions. The most promising strategy is not to replace the physical model with a neural network, but to train a surrogate model on high-precision simulations while preserving physical constraints [128,129,130,131,132,133,134,135,136].

An AI surrogate can approximate the fluence map in milliseconds, whereas the original model requires minutes or hours. This makes it possible to explore thousands of light-guide configurations, analyze uncertainty, and adaptively replan during the procedure. However, this advantage is valid only within the scope of the training set. If the model is trained on a limited range of absorption coefficients, tumor sizes, or source geometries, extrapolation may produce maps that look physically plausible but are in fact incorrect. For this reason, physics-informed loss functions, conservation-of-energy checks, comparison with independent simulations, and phantom measurements should be used. Prediction uncertainty should be reported alongside the calculated dose, and cases that fall outside the training space should be automatically sent for a full physical calculation [57,128,136].

### 6.3. Reconstructing Tissue Optical Properties Using Machine Learning

Pre-operative values of absorption and scattering coefficients are often taken from the literature or from averaged measurements, although inter-patient and intra-tumor variability can be considerable. In interstitial PDT, optical properties can be estimated from signals between inserted sources and detectors. The inverse problem is nonlinear and often ill-conditioned: different combinations of parameters can produce similar measurements. Yassine et al. showed that machine learning models are capable of reconstructing patient-specific optical parameters from synthetic dosimetric data and using them to re-optimize the power of light guides in near-real time [124].

The key condition for clinical applicability is training on a realistic error distribution: photodetector noise, uncertainty in coordinates, tissue heterogeneity, differences in source spectrum, and light-guide calibration. The model should not only output point estimates of μa and μs′ but also estimate confidence intervals and the correlation between parameters. A hybrid approach is useful, in which ML provides an initial estimate for subsequent physically grounded optimization. Such a scheme speeds up the inverse problem while preserving the verifiability of the result and making it possible to reject solutions that are incompatible with the measurements [124,137].

### 6.4. Optimization of the Placement and Power of Light Guides

The task of interstitial planning covers selecting the number of light guides, their coordinates, the length of the active diffuser, orientation, power, and exposure duration. The objective function should maximize the proportion of the tumor receiving therapeutic irradiance and fluence, while at the same time limiting the excessive exposure of vessels, nerves, airways, and other critical structures. A simple uniform grid is not always optimal for irregularly shaped tumors. Modern systems use convex optimization, evolutionary algorithms, Bayesian optimization, or sequential optimization after each geometric change. In a clinically oriented study, Oakley and co-authors demonstrated joint optimization of irradiance and fluence for interstitial PDT on a malignant obstruction of the central airways using finite-element solvers [129,137,138,139,140].

Optimization is multi-criteria: maximum tumor coverage may conflict with limits on peak irradiance, procedure duration, the number of punctures, and the technical accessibility of trajectories. For that reason, a single scalar metric is not always adequate. Pareto optimization generates a set of non-dominated solutions from which the clinician chooses according to the clinical priority. For example, one plan may provide higher minimum coverage at the cost of an additional light guide, while another may offer slightly lower homogeneity with less invasiveness. The algorithm must account for discrete decisions, such as the number and type of diffusers, as well as continuous parameters of power and time. Bayesian optimization is particularly useful when physical simulation is computationally expensive, as it selects the next configuration on the basis of expected improvement and uncertainty. Genetic algorithms are more flexible under complex constraints but require a large number of computations and careful verification of reproducibility [129,130,141].

For personalized PDT, plan robustness is important. Even a mathematically optimal configuration may become unsatisfactory if the needle shifts by 2–3 mm, the source power deviates, or there is a segmentation error. Robust optimization evaluates not one ideal scenario, but an ensemble of possible realizations, and maximizes the minimum guaranteed coverage. The open tools FullMonte and PDT-SPACE have demonstrated the feasibility of scalable planning that takes individual geometry into account, and later studies extended the analysis to cylindrical diffusers, the spatial heterogeneity of the photosensitizer, and parameter uncertainty [130,131,132,141]. AI is useful here for the rapid ranking of scenarios and identifying the most sensitive variables, but the final plan must undergo physical and clinical verification.

### 6.5. Surface PDT and Dynamic Light Planning

For dermatological and intraoperative surface PDT, automation may include three-dimensional scanning of the anatomical area, construction of a digital surface, and calculation of local irradiance with allowance for distance and angle. Wang and co-authors developed a system with a 3D scanner that optimizes lighting uniformity, and later demonstrated a three-dimensional image-guided platform with dynamic planning and light dose monitoring [125,126]. Such systems are especially useful for large, curved, or multiple lesions, where manual positioning of the lamp creates marked heterogeneity.

The control algorithm can regulate individual segments of an LED array, the trajectory of a scanning source, or the exposure of different areas. At the same time, it is necessary to distinguish the geometric uniformity of light from the uniformity of the photochemical effect. An area with greater pigmentation, a lower PpIX concentration, or pronounced hypoxia may require a different regime, despite having the same measured fluence. Therefore, promising systems should combine 3D geometry with a fluorescence map of the photosensitizer, reflectance spectroscopy, and oxygenation indicators [58,60,126].

### 6.6. Oxygen, Photobleaching, and Photochemically Effective Doses

A high fluence rate can rapidly deplete the available oxygen and shift part of the exposure into a mode with low ROS generation efficiency. Reducing power, fractionation, or pauses allow the oxygen content to recover partially through perfusion and diffusion. The optimal regime depends on the photosensitizer, vascular response, and initial oxygenation; the universal recommendation that “the lower the power, the better” is also incorrect, because an excessively long procedure may intensify sensitizer clearance, discomfort, and organizational constraints. The computer model should link light transport with the kinetics of oxygen, the photosensitizer, and the singlet oxygen produced [5,6,139].

Fractionated PDT is a natural sequential decision-making problem. After the first light fraction, one can assess the rate of photobleaching, the drop in oxygenation, and the vascular response, and then choose the duration of the pause and the parameters of the next stage. In metronomic PDT, low-intensity irradiation continues for a long time and requires monitoring of the accumulated dose, source stability, and the tissue’s physiological adaptation. AI can build a short-term forecast of oxygen recovery and compare alternative regimens by the expected reacted singlet oxygen, procedure duration, and risk of a thermal effect. However, the model must take into account that measured oxygen reflects the balance between delivery and consumption, not merely photochemical activity. Perfusion changes after vascular injury can both temporarily improve oxygen supply and cause persistent ischemia. Therefore, training data should contain time series rather than only initial and final points [58,138,139].

Photobleaching is used as a component of implicit dosimetry, as a decrease in fluorescence may reflect the course of the photochemical reaction. However, the signal depends on concentration, detection geometry, blood volume, and the formation of photoproducts. Therefore, it cannot be interpreted in isolation. Combined monitoring of chlorin e6 fluorescence, hemoglobin oxygenation, and backscatter made it possible to track photobleaching and vascular changes without interrupting illumination [58]. In pleural PDT, real-time systems were developed that combine light measurements, Photofrin concentration, and a reacted singlet oxygen model, illustrating the shift from nominal fluence to multi-component dosimetry [133,134,140].

### 6.7. Adaptive Control and the Digital Twin of PDT

Adaptive PDT involves a closed loop: sensors record light, fluorescence, oxygen, blood flow, or temperature; the algorithm estimates the current photodynamic dose and predicts subsequent dynamics; the control system changes the power, active light guides, duration, or pause. Unlike a static plan, this approach responds to changes caused by the therapy itself. The algorithm may use model predictive control, Bayesian updating, or reinforcement learning; however, training directly on patients is ethically unacceptable without a safe simulation environment and strict action constraints.

**Figure 5:**
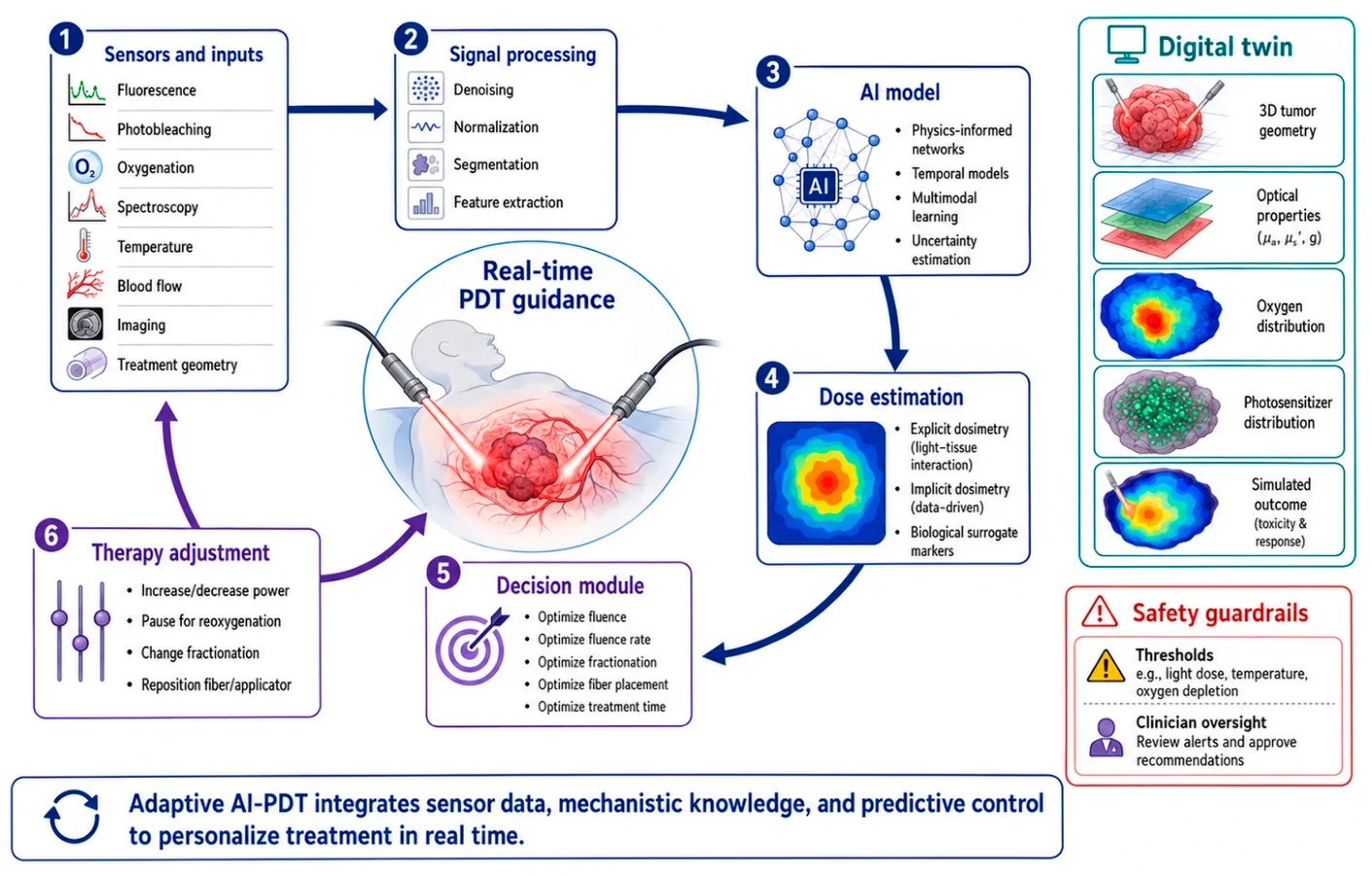
Adaptive AI-controlled photodynamic therapy.

The choice of control algorithm depends on how fully the system is observed. If the tissue state is well described by measurable parameters, model predictive control is suitable: at each step, several future trajectories are calculated, a safe action is selected, and after a new measurement, the forecast is recalculated. For a partially observable state, preference should be given to Bayesian filters or recurrent models that estimate hidden variables, such as the local oxygen concentration outside the sensor zone. Reinforcement learning can optimize a complex sequence of interventions in a digital phantom, but before clinical use, the control policy must be constrained to permissible power ranges and tested on rare adverse scenarios. The algorithm must not be allowed to increase exposure merely to improve a surrogate metric while ignoring the risk of damage to normal tissues [124,129,136]. An example of a PDT scheme, controlled by AI, is demonstrated in Figure 5.

A digital twin of PDT should integrate segmented anatomy, the distribution of optical parameters, photosensitizer concentration, perfusion, oxygen kinetics, and a damage model. After each measurement, its state is updated, and the next regimen is chosen on the basis of the forecast. In the near term, a universal digital twin is less realistic than modular models for a specific clinical task: for example, power control during pleural PDT or replanning interstitial irradiation after refining the optical properties. Clinical implementation requires predefined safe limits, logging of all changes, and the ability to switch immediately to manual control [7,124,133].

### 6.8. Validation, Standardization, and Clinical Readiness

Uncertainty should be treated as an independent planning outcome. Its sources include variability in optical properties, segmentation errors, source misplacement, imperfect calibration, heterogeneity in photosensitizer distribution, and structural model error. In practice, it is useful to build probabilistic maps: for example, the probability of achieving the minimum therapeutic fluence at each point and the probability of exceeding the permissible level in normal tissue. Sensitivity analysis shows which parameters must be measured in each individual patient and which may be represented by a population distribution. This approach prevents false precision and enables the clinician to compare conservative and more aggressive plans on the basis of a quantitative risk assessment [130,131,132,135,141].

Validation of an AI dosimetry system should cover several levels. At the computational level, it is compared with a reference model on independent geometries and parameter ranges. This is followed by measurements in optical phantoms with known properties, ex vivo and in vivo studies, retrospective verification on clinical data, and finally a prospective study. Errors in segmentation, source positioning, power calibration, and optical parameter recovery should be assessed separately. Mean error is not enough: worst-case scenarios, minimum tumor coverage, and maximum dose to organs at risk are critical [57,129,130,131,132,141].

The temporal stability of the model should also be tested separately. Updates to the laser system, software, optical fibers, or imaging protocol can alter the input data distribution and reduce accuracy without any obvious failure. Control phantom measurements, monitoring for drift, periodic recalibration, and predefined criteria for re-validation are therefore necessary. The algorithm version and calculation parameters should be stored together with the procedural protocol so that the result can be reproduced and audited [57,125,126].

A standardized report should include the wavelength, spectral bandwidth, irradiance, fluence, fractionation mode, source geometry and calibration, tissue optical parameters, photosensitizer concentration, drug–light interval, method used to monitor oxygen and photobleaching, calculation algorithm, and estimate of uncertainty. For ML, the training range, data distribution, external validation, and model rejection criteria need to be described. A clinically mature system should not merely produce an attractive light-distribution map, but demonstrate improved coverage, a reduced risk of damage to normal tissue, or a more accurate prediction of response. Thus, the role of AI is to accelerate and integrate the physically measurable components of PDT, not to replace photochemical dosimetry with an opaque statistical indicator [26,27,28,29,30,31,32,57,124]. Information on AI methods and computational modeling in photodynamic therapy planning and dosimetry is gathered in Table A4.

## 7. AI-Based Visualization, Spectroscopy, and Monitoring PDT in Real Time

### 7.1. Visualization as an Origin of the Dynamic Biomarkers of PDT

Visualization in PDT has two different functions: it characterizes the original state of biological objects and registers changes that appear in the cancer during activation of the photosensitizer. For AI–PDT applications, imaging evidence should be interpreted according to three temporal stages: pre-treatment imaging for lesion characterization, treatment planning, and baseline assessment; intra-treatment imaging for monitoring photosensitizer distribution, oxygenation, photobleaching, perfusion, and other dynamic treatment variables; and post-treatment imaging for assessing biological response, tissue damage, residual disease, and longer-term outcomes. Lesion identification and tumor-boundary segmentation belong primarily to photodynamic diagnosis or treatment planning and should not be treated as equivalent to PDT efficacy monitoring. AI converts these data from mostly qualitative observations into reproducible quantitative characteristics. On the clinical level, the YOLOv3 algorithm was automatically used to calculate live and dead cells after PDT, and Cellpose allows for evaluation of the surface, shape, and proliferation dynamics of individual cells during the observation process [66,142].

In this context, the input consists of serial cellular images acquired after treatment, the relevant time point is post-treatment, and the principal endpoint is the quantitative cellular response rather than lesion diagnosis.

The principal advantage of temporary images is the ability to detect early responses before forming irreversible macroscopic necrosis. However, the cadre sequence is not an independent observation. Cells belong to one culture, images to one field of vision, and serial images to one experiment. Because of this separation between learning and testing, samples must be present at the level of biological experiments, animal or patient, not a separated cadre. Otherwise, the model remembers background characteristics and the characteristics of the microscope and samples. In addition, automatic segmentation must be accompanied by an evaluation of quality masks and manual control of hard cases. Because of cell fusion, nuclear fragmentation, and loss of contrast after oxidative damage, systematic changes in the algorithm can cause errors [66,142].

### 7.2. Fluorescent Visualization of the Photosensitizer and Photobleaching

The fluorescence of the photosensitizer is a natural diagnostic signal, allowing the evaluation of accumulation, localization, and changes in concentration during the irradiation interval. Signal intensity does not depend only on the concentration of the molecule, but also on depth, hemoglobin adsorption, the scattering geometry of registration, quantum yield, and aggregation. A two-component approach uses different penetration components of light and the signal ratio to evaluate the depth of chlorine photosensitizer localization. Analytics, numbers, and clinical research show that such monitoring can characterize photosensitizer distribution before treatment and its photobleaching during irradiation [143,144].

Accordingly, fluorescence imaging can provide a pre-treatment input describing photosensitizer accumulation and spatial distribution, whereas intra-treatment fluorescence provides time-resolved information on photobleaching and redistribution. The corresponding endpoints are baseline drug localization before irradiation and dynamic photochemical response during treatment.

AI is very useful during the transition from one-dimensional spectra to spatial–time maps. The model can consider tissue autofluorescence and product spectral overlap, optical property changes, and patient movement. Instead of a direct model of the efficacy of a decrease in intensity, the hybrid model is preferable. It connects fluorescence and the parameters of light transfer, photosensitizer concentrations, and oxygen kinetics. Photobleaching is informative, but not a universal surrounding marker: its connection to the generation of different ROS depends on the chemical structure, microenvironment, and excitation mode. Hence, the algorithm must provide information about uncertainty and point to the conditions where calibration dependence loses its validity [58,143,144].

### 7.3. FLIM and Metabolic Visualization

Fluorescence lifetime imaging microscopy (FLIM) measures the lifetime of the excited state, which depends less on fluorophore concentration and excitation power than common visualization by intensity. In oncology, metabolic contrast first forms NAD(P)H and FAD: the difference between free and bound forms mirrors the difference between glycolysis and oxidative phosphorylation. PDT quickly disrupts metabolic mitochondrial function, oxidation–reduction equilibrium changes, and membrane potential. Due to this, FLIM can detect the response earlier than a standard test of vitality. A modern review highlights a promising combination of FLIM with deep learning for automatic classification of tissues and therapeutic evaluation of response [145,146,147].

For PDT monitoring, FLIM is primarily relevant to intra-treatment and early post-treatment assessment. Its inputs include fluorescence lifetime and metabolic parameters; the time points are baseline, during treatment, and shortly after irradiation; and the endpoint is early metabolic response rather than tumor-boundary identification.

Machine learning solves two problems of FLIM: long accumulation of photons and the complexity of multiparameter approximation. Neural network models can recover data maps from lifetime from low-signal data, facilitating processing and highlighting the composition of parameters that are not obvious when analyzing the average lifetime. Due to this, the algorithm has no hidden physics of changes. Validation must be compared with time-correlated single-proton counting, instrument response function control, and checkout on independent equipment. Recent work has shown the possibility of metabolic changes in cancer cells using two-factor FLIM and machine learning, and FLIM for the evaluation of cancer boundaries under intraoperative conditions [148,149,150].

The latter application represents intraoperative lesion delineation or auxiliary diagnosis and should therefore be distinguished from FLIM-based monitoring of PDT efficacy.

Of particular interest is the simultaneous registration of the qualitative phase of visualization and FLIM. QPI characterizes dry mass, surface, optical thickness, and morphological dynamics. The FLIM mirrors the metabolism and intracellular accumulation of the photosensitizer. On HeLa and A549 cultures, this multimodal system is used to observe morphological and physiological changes after the influence of Radachlorin. For AI, this creates more space for consequential signs: the model can be different for early metabolic dysfunction versus later loss of integrity. It can conclude differences between cell lines [151,152].

Here, QPI and FLIM provide complementary post-treatment inputs describing morphology and metabolism, with the endpoint defined as the temporal cellular response to PDT.

### 7.4. Raman and Diffuse Reflectance Spectroscopy

Raman spectroscopy provides non-labeling information about proteins, lipids, nucleic acids, and metabolites, but it forms high-dimensional spectra with correlated variables and a pronounced background. Machine learning is used to remove the background, which can be fluorescent. It can also be used for decreasing dimensions, tissue classification, and the identification of spectral biomarkers of response. However, a direct clinical set of Raman–AI and PDT for PDT is present in very small amounts. It was shown that spectroscopy and ML can register early molecular changes of the tumor microenvironment even before a pronounced reduction in the tumor. This proves the validity of the early-monitoring approach for photodynamic damage and immune responses [153,154].

In this application, Raman spectra are acquired mainly during early post-treatment monitoring, with molecular spectral features as inputs and photodynamic damage or immune response signatures as prediction endpoints.

Diffuse reflectance spectroscopy evaluates absorption, scattering, hemoglobin oxygenation, and perfusion. For PDT, these parameters are critical because they simultaneously influence light delivery and oxygen availability. Spectral inversion is rarely determined by the task, and ML can accelerate parameter recovery. ML can facilitate parameter recovery if the training data are generated by physically realistic models and phantom experiments. In neuro-oncology, we have accumulated a lot of experience and touched upon the application of AI in fluorescence, Raman, diffuse reflectance, and other optical spectra. However, to transfer this approach to PDT, we should take into account the change in spectra directly during the learning process and separate it from heating, bleeding, and moving [137,155,156].

Diffuse reflectance spectroscopy can be used in pre-treatment to estimate baseline optical and vascular properties. It can be used intra-treatment to monitor changes in oxygenation and perfusion. The corresponding endpoints should therefore be defined separately for baseline characterization and dynamic treatment monitoring.

Surface-enhanced Raman spectroscopy (SERS) increases sensitivity enabled by plasmonic nanostructures and can be combined with a photosensitizer in a single theranostic platform. Such a system is able to confirm delivery of nanoconstructions, recognize the molecular microenvironment, and monitor oxidation products. Machine learning methods are used for the deconvolution of overlapping peaks, compensation for variability, and building substrate-to-substrate spatial maps. However, they are not a prerequisite for accurate SERS analysis. Due to this, plasmon amplification can alter heat generation; the quantum yield of the photosensitizer and diagnostic and therapeutic components must be calibrated separately, but not reviewed as independent modules [157,158].

### 7.5. Radiomics and Spatial Assessment of Responses

CT, MRI, and PET radiometry extracts from medical images show quantitative characteristics of shape, intensity, and texture, potentially reflecting necrosis, fibrosis, vascularity, and heterogeneity. Analysis of the photodynamic priming of adenocarcinomas in the pancreas in clinical research allows determination of necrosis zones and changes in surrounding tissues. The latter cannot be viewed as just simple changes in the cancer diameter [159]. This sample is important because it demonstrates changes from optical registration to the evaluation of deep-tissue reactions in clinical data.

Radiomic analysis should be separated into pre-treatment and post-treatment tasks. Pre-treatment CT, MRI, or PET features can characterize lesion geometry and heterogeneity for planning or prognosis, whereas post-treatment imaging can quantify necrosis, fibrosis, vascular changes, and other response endpoints. Tumor segmentation performed before treatment is therefore a planning or diagnostic task, not evidence of PDT efficacy monitoring.

Radiometric monitoring must be standardized using scanning, reconstruction, and segmentation parameters, as well as intensity normalization. Changing the CT protocol can have an influence on texture signs and therapy. For PDT, the most promising perspective is delta-radiomics for analyzing different signs before and after the procedure. However, small cohorts allow restricted numbers of samples, nested cross-validation, and external validation. Spatial alignment of maps of the fluorescence, light doses, and CT/MRI can establish a causal link between local exposure and damage, not only the statistical correlation but also between the original image and the result.

### 7.6. Multimodal Integration and Self-Registering Theranostic Systems

A single modality cannot describe all PDT chains. Fluorescence characterizes photosensitizers; diffuse reflectance characterizes optical properties and blood; FLIM characterizes metabolism; QPI characterizes morphology; Raman characterizes molecular composition; and CT/MRI characterizes anatomical results.

These modalities should be integrated with explicit temporal labels: pre-treatment inputs characterize anatomy, optical properties, baseline metabolism, and photosensitizer distribution; intra-treatment inputs monitor photobleaching, oxygenation, perfusion, and evolving photochemical changes; and post-treatment inputs quantify metabolic, morphological, molecular, and anatomical response.

Multimodal learning can be used through different signs, independent encoders with late fusion, or attention mechanisms. The preferred architecture saves modality-specific information and allows for the absence of part of the data. Otherwise, a system trained on the full set of measurements becomes unsuitable for clinical settings, where some sensors are unavailable or yield signals of insufficient quality [151,155,160].

A promising area of research involves self-reporting photosensitizers and nanotheranostics. They can have an optical signal that changes during cell death, drug release, and activation in the microenvironment. The PDT agent, which is able to detect cell death in real time, was described in 2025. Such a construction can provide a more specific outcome than a nonspecific decrease in intensity. However, we must prove that the reporter signal is quantitatively related to the endpoint, does not alter phototoxicity, and requires calibration in vivo. AI can integrate the kinetics of multiple channels and recognize response patterns, but it does not replace chemical validation of the mechanism [160,161].

### 7.7. Hyperspectral Imaging, OCT, and Spatial Registration

Hyperspectral imaging combines spectroscopic sensitivity with spatial resolution: each pixel contains a spectrum, enabling simultaneous mapping of the photosensitizer, hemoglobin, autofluorescence, and potential oxidation products. For PDT, this is particularly important when drug accumulation is heterogeneous, and the lesion geometry is complex. However, the data volume is large, neighboring spectral channels are correlated, and the signal is sensitive to lighting and movement. Convolutional and spectral–spatial neural networks can perform segmentation, classification, and unmixing, but they must be trained on data with physically correct calibration of reflectance and wavelength. A useful strategy is self-supervised pre-training on unlabeled cubes, followed by fine-tuning on a small set of confirmed regions [154,155,160].

Hyperspectral imaging can contribute to pre-treatment mapping of lesion geometry and photosensitizer distribution and to intra-treatment monitoring of hemoglobin-related and spectral changes. Segmentation and classification outputs should be reported separately from treatment response endpoints.

Optical coherence tomography (OCT) produces microstructural images of tissue to a depth of several millimeters and can measure epithelial thickness, edema, vascular changes, and architectural disruption. In dermatological PDT, OCT can complement surface fluorescence: the former shows the structural effect, while the latter shows the distribution of the photosensitizer. Neural network segmentation of layers and vessels reduces operator variability; however, therapeutic changes may disturb the usual anatomy and reduce the accuracy of a model trained on baseline images. It is therefore necessary to include early and late post-therapy images, and to test robustness to speckle noise, differences between scanners, and optical artefacts [142,155].

Thus, baseline OCT segmentation is principally a pre-treatment characterization task, whereas changes in tissue structure in early and late post-treatment OCT images represent response-monitoring endpoints.

For causal interpretation, multimodal maps must be registered in a single coordinate system. Surface fluorescence, the hyperspectral cube, OCT, and subsequent histology have different resolutions and deform during tissue processing. Registration errors can create a false correlation between high dose and damage. Deformable registration algorithms should be validated against anatomical landmarks, and transformation uncertainty should be included in statistical analysis. In clinical studies, it is advisable to preserve the original light-field coordinates and camera position in order to link local features across pre-treatment, intra-treatment, and post-treatment stages [126,143,159].

### 7.8. Longitudinal Models, Transferability, and Domain Shift

The temporal dynamics of PDT cannot be reduced to the independent classification of individual frames. Early fluorescence decline, a short-term change in oxygenation, slowing of cell movement, and subsequent fragmentation form a consistent pattern. Recurrent neural networks, temporal convolutional networks, and transformers can take account of the order and intervals of observations, predicting the likelihood of irreversible damage. For small datasets, models with a limited number of parameters and predefined biophysical features are preferable. Time points after treatment has finished must not be randomly included among the features of a model intended to make decisions during the procedure, as this creates a hidden leak of future information [66,142,151].

Accordingly, models intended for intra-treatment decision support must use only information available up to the current treatment time point, whereas post-treatment endpoints should be reserved for model validation or outcome prediction.

Domain shift arises when the photosensitizer, wavelength, camera, objective lens, cell line, or clinical center changes. A model may retain high internal accuracy and perform markedly worse on new equipment. To detect shift, feature-distribution monitoring, uncertainty-aware prediction, and test phantoms are used. Transfer learning and domain adaptation can reduce the need for full labeling of a new dataset, but adaptation must be carried out without mixing the test cohort with the training cohort. In a clinical system, criteria for re-validation must exist after hardware or protocol updates [15,145,154,155].

Interpretability is especially important for spectral models. An attention map or SHAP values must be linked to biologically plausible ranges: bands of lipids, proteins, porphyrins, NAD(P)H, or hemoglobin. If a classifier uses an image edge, noise from a particular detector, or a spectral channel with no physical signal, this indicates confounding. Permutation tests, modality ablation analysis, and checking against synthetic artefacts help establish whether the model truly relies on therapeutically meaningful features. Such validation is necessary before the result is used to change light exposure [153,154,155,156,160].

### 7.9. Requirements for Real-Time Operation and Clinical Validation

Real-time monitoring means not simply rapid image acquisition, but a complete cycle: registration, artefact correction, segmentation, prediction, uncertainty estimation, and outputting a decision with latency acceptable for protocol changes. For surface PDT, this may be seconds; for interstitial PDT, it may be minutes. The model must detect movement, detector saturation, loss of contact, and values outside the training range. When quality is low, it should not make automatic decisions. Forward-looking reviews of AI–PDT regard such feedback as the basis for the transition to adaptive personalized therapy, although most published studies are still at the proof-of-concept stage [15,17].

Real-time systems therefore belong specifically to the intra-treatment stage. Their inputs are contemporaneous imaging, spectral, oxygenation, or perfusion measurements; the time point is during irradiation; and the clinically relevant endpoint is an actionable estimate of treatment state or the need for protocol adaptation.

Clinical evaluation must assess not only Dice, accuracy, or AUC, but also the system’s impact on the procedure: changes in exposure, prevention of under-treatment, toxicity, and local control.

Margin delineation should be reported separately as a diagnostic or treatment-planning endpoint, whereas changes in exposure, prevention of under-treatment, toxicity, and local control represent PDT treatment or outcome endpoints.

Prospective protocols with pre-specified thresholds and an independent reference standard are required. For temporal data, calibration of the prediction should be checked at each stage, and for multimodal systems, robustness should be checked in the event of a missing channel. Output images, preprocessing, model versions, and equipment parameters must be stored for audit purposes. Only when these conditions are met does AI visualization become a measurable component of dosimetry, rather than a post hoc illustration of therapeutic effect [15,66,142,155,159].

AI-compatible methods for monitoring the response to photodynamic therapy are systematized in Table A5.

## 8. Prediction of Biological and Clinical Outcomes of Photodynamic Therapy

### 8.1. Defining Therapeutic Response and the Hierarchy of Endpoints

Predicting the outcome of PDT first requires a strict definition of what counts as a response. At the preclinical level, researchers use the generation of reactive oxygen species, reduced metabolic activity, clonogenic survival, the proportion of apoptotic, necrotic, or ferroptotic cells, spheroid regression, and delayed tumor growth. However, these measures reflect different temporal and biological levels and are not interchangeable. A rapid fall in a viability assay signal may not coincide with loss of proliferative capacity, while pronounced local necrosis does not necessarily ensure durable tumor control. AI models should therefore be trained on predefined endpoints, take the time of measurement into account, and distinguish between the immediate photochemical effect, the early cellular response, and the late tissue outcome [162,163,164].

In clinical studies, the target variables are complete or partial response, local control, recurrence, recurrence-free and overall survival, pain severity, functional and cosmetic outcomes, and toxicity. For dermatological lesions, clinical and histological clearance of the lesion is important; for interstitial PDT, the volume of necrosis and preservation of critical structures are important; for ophthalmology, anatomical parameters and visual acuity are important. Grouping heterogeneous outcomes into the binary category of “responder/non-responder” simplifies analysis but may hide clinically significant differences. Multi-target models are preferable as they simultaneously assess the probability of response, severe complications, and repeat treatment [162,165,166].

The minimally clinically important change should also be fixed in advance. A statistically significant reduction in lesion volume may not improve function or quality of life, whereas a moderate anatomical change is sometimes accompanied by a marked symptomatic effect. Models trained only on surrogate laboratory measures should be regarded as hypothesis-generating until the link with patient-centered outcomes is confirmed [162,165].

### 8.2. Clinical, Photochemical, and Procedural Predictors

The minimum set of features for clinical prediction should combine patient, disease, and procedural characteristics. These include age, comorbidities, histological type, stage, lesion size and depth, previous treatment, photosensitizer concentration, drug-to-light interval, wavelength, fluence, fluence rate, irradiation geometry, and measured oxygenation. Particularly important are parameters that change during the procedure: photobleaching, fluorescence dynamics, perfusion, and oxygen replenishment. Including these makes it possible to move from static prediction to an updated risk assessment, in which the probability of response is recalculated as monitoring data arrive [165,166,167,168].

It is necessary to distinguish between prognostic and predictive biomarkers. A prognostic feature is associated with the natural course of the disease regardless of treatment, whereas a predictive one indicates the likelihood of benefit from a specific PDT regimen. For example, a large tumor volume may be a general adverse factor, whereas a low concentration of photosensitizer or pronounced hypoxia may be a factor that directly limits the photodynamic effect. A causal interpretation is particularly important if the model is to recommend changes to fluence rate, fractionation, or combination therapy, rather than merely classifying patients [129,162,167].

**Figure 6:**
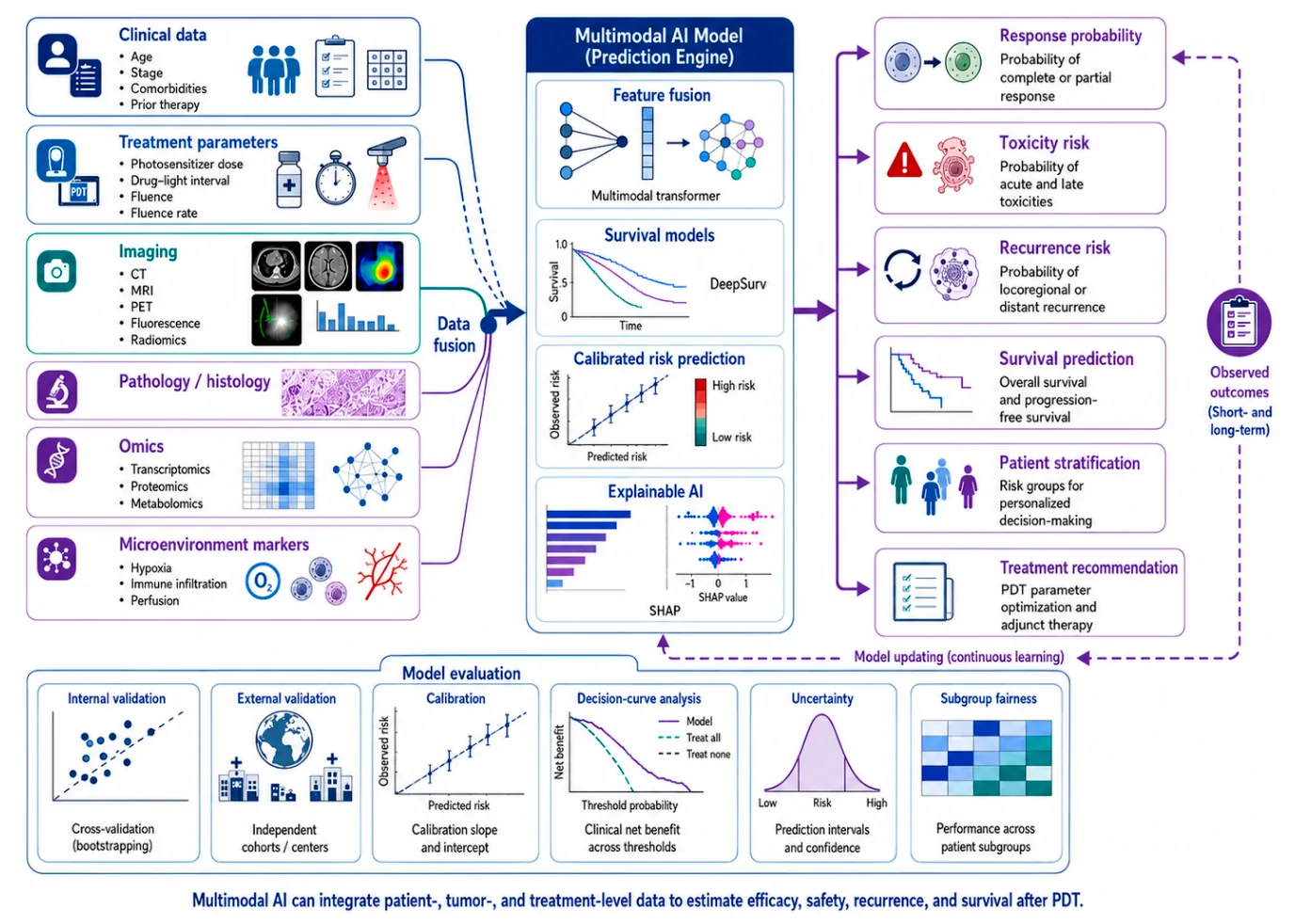
Multimodal prediction of patient outcomes after PDT.

When designing the feature space, mechanical inclusion of all available variables should be avoided. Some parameters are derived from others: fluence is determined by power and time, while integral fluorescence depends simultaneously on the photosensitizer concentration, optical attenuation, and the detection geometry. Redundancy increases coefficient instability and complicates interpretation. A rational strategy includes predefined clinical features, mechanistically grounded photochemical parameters, and a limited set of interactions. For time-series data, useful measures are the photobleaching rate, the area under the oxygenation curve, and the oxygen recovery time between fractions. These aggregates should be computed exclusively within the training set in order to prevent information leakage [129,167,168]. The conception of a multimodal outcome prediction system is described in Figure 6.

### 8.3. Visual Biomarkers and Spatial Heterogeneity

Radiomics and quantitative optical imaging make it possible to describe spatial heterogeneity, which is poorly captured by a single measurement of tumor size. CT and MRI texture features, perfusion and diffusion parameters, vascular architecture, the depth of photosensitizer accumulation, and the distribution of photobleaching may reflect stromal density, necrosis, hypoxia, and light availability. Delta-radiomics is particularly informative: it analyses changes in features between the baseline scan, an early time point after PDT, and subsequent follow-up. In a study of photodynamic priming in pancreatic adenocarcinoma, quantitative CT features made it possible to detect tissue changes beyond the zone of direct ablation, demonstrating the potential of imaging as an early surrogate marker [67,159].

For proper training, data must be split at the patient level, not at the level of individual slices or images; otherwise, images from the same patient may end up in both the training and test sets at the same time. The influence of the scanner, reconstruction protocol, voxel size, and manual segmentation must also be controlled. With small cohorts, predefined features, regularization, and nested cross-validation are preferable; deep networks without external validation carry a high risk of overfitting. Spatial maps of uncertainty may be more clinically useful than a single numerical probability value, as they show the areas where the prediction is driven by a lack of data or ambiguous segmentation [66,67,159].

Multimodal fusion can be carried out at early, intermediate, or late stages. Early fusion forms a single feature matrix but requires strict normalization and becomes more difficult when modalities are missing. Intermediate fusion trains separate encoders for images, spectra, and clinical data, then combines their latent representations. Late fusion averages or weights the predictions of independent models and is usually more robust in small clinical cohorts. The choice of architecture should be determined not by technological novelty, but by sample size, data completeness, and the intended use case [66,67,159].

### 8.4. Transcriptomics, Proteomics, and Metabolic Signatures

Omics data make it possible to move from phenomenological prediction to mechanism-based response models. RNA-seq after 5-ALA PDT reveals changes in heat-shock genes, the extracellular matrix, focal adhesion, TGF-β signaling, and stress responses. In other models, integrated transcriptomic analysis demonstrates the remodeling of apoptosis, autophagy, inflammation, antioxidant defense, and repair pathways. For building a predictive panel, it is important not only to consider differentially expressed genes after irradiation, but also baseline signatures that characterize tissue sensitivity in advance [163,164,169].

Potential features include the expression of heme biosynthesis enzymes, transporters and efflux proteins, HIF-1α, NRF2, HSP90, antioxidant enzymes, and components of the xCT–GSH–GPX4 and FSP1–CoQ10 systems, as well as markers of immune antigen presentation. Metabolomics can assess glutathione, NADPH, lactate, porphyrins, and the availability of polyunsaturated lipids. As the number of molecular features far exceeds the number of samples, filtering by biological pathways, sparse models, and independent validation in another cohort are necessary. Otherwise, the model memorizes the laboratory batch rather than a robust mechanism of sensitivity [166,169,170,171].

Missing omics and clinical data cannot automatically be assumed to be random. The absence of a biopsy, spectroscopic measurement, or control scan is often linked to disease severity, treatment logistics, or early progression. Such informative missingness carries a prognostic signal in itself but can also amplify bias. Multiple imputation, models with availability masks, and complete-case analysis should be compared, and the proportion of missing values should be reported for each feature. For omics panels, it is necessary to verify the stability of the selected genes under resampling and changes in the normalization algorithm [163,164,165,166,169].

### 8.5. Predicting the Type of Cell Death and Resistance

PDT can induce apoptosis, necrosis, autophagy-associated cell death, pyroptosis, and ferroptosis; the balance between these programs depends on the localization of the photosensitizer, the intensity of damage, oxygen availability, and the cell’s redox buffering capacity. Mitochondrial damage is more often associated with activation of caspase-dependent apoptosis, massive membrane oxidation with necrosis, and the accumulation of lipid peroxides when GPX4 or SLC7A11 is reduced during ferroptosis. Accordingly, prediction should rely on a joint analysis of morphology, the temporal dynamics of ROS, membrane potential, caspases, lipid peroxidation, and molecular markers, rather than on a single test [170,171,172,173].

Resistance may arise as a result of hypoxia, increased efflux of the photosensitizer, activation of NRF2, elevated glutathione content, autophagic disposal of damaged organelles, and membrane repair. Studies of ferroptosis show that inhibition of GPX4, xCT, or FSP1 increases sensitivity to photo-oxidation and, at the same time, may enhance the immunogenicity of cell death. These features are suitable for patient stratification and selection of combinations; however, their predictive value should be validated independently of demonstrating the mechanism in a cell line [170,172,173,174].

### 8.6. Immune Response as a Long-Term Outcome

Local tumor destruction does not fully characterize the therapeutic value of PDT. Photo-oxidative stress can cause calreticulin exposure, ATP and HMGB1 release, dendritic cell maturation, and the formation of a CD8+ T-cell response. Therefore, models of long-term control should include indicators of immunogenic cell death, baseline immune infiltration, the proportion of Tregs, the state of myeloid cells, and checkpoint expression. Single-cell sequencing has shown that combining PDT with the PD-1 blockade remodels the microenvironment in oral carcinogenesis, reducing the proportion of Tregs and S100A8-positive populations and enhancing the therapeutic immune response [165,166,175].

An experiment with HSP90-targeted PDT and the PD-L1 blockade demonstrated a systemic and abscopal effect, and a CXCR3-associated gene signature was proposed as a potential biomarker. For AI, this means the need to model not only local tumor volumes, but also the likelihood of systemic immune activation. Graph-based and spatial models that capture interactions between tumor, immune, and stromal cells are promising. At the same time, immune endpoints are sensitive to animal species, microbiota, sampling time, and the antibodies used, which limits the direct transfer of preclinical classifiers into the clinic [168,175,176].

### 8.7. Prediction of Toxicity, Recurrence, and Survival

A clinically useful system must predict benefit and harm at the same time. Significant complications include damage to normal tissue, edema, pain, inflammation, necrosis, strictures, and prolonged cutaneous photosensitivity. Risk depends on the distribution of light and the photosensitizer, proximity to critical structures, vascular response, and the tissue’s capacity for repair. In dermatology, skin phototype and anatomical site must be taken into account; in the brain, the likelihood of neurological deficit; and during interstitial procedures, damage to blood vessels and hollow organs. Multi-objective models can identify Pareto-optimal regimens, in which an increase in the probability of complete response is not accompanied by an unacceptable rise in toxicity [162,167,177].

For time-to-event outcomes, Cox regression, random survival forests, gradient boosting, and neural survival models are applicable. They must account for censoring, competing risks, and time-varying features. Assessment of quality is not limited to the concordance index or ROC–AUC: Brier score, calibration curves, time-dependent AUC, and decision-curve analysis are also required. Even a model that discriminates well between patients is dangerous if it systematically overestimates the absolute probability of recurrence. For rare complications, precision–recall characteristics and analysis of sensitivity to class imbalance are important [26,67,177].

After implementation, the model requires monitoring for changes in calibration. New photosensitizers, light sources, imaging protocols, and changes in patient selection criteria create temporal dataset shifts. Periodic retraining without controls may compromise safety, so updates should be accompanied by versioning, independent verification, and comparison with the original locked model. It is advisable to define, in advance, the thresholds at which the system stops issuing a personalized recommendation and reports that the data have moved outside the domain of applicability [26,177,178].

### 8.8. Validation and Clinical Utility of Predictive Models

The most serious problem in AI–PDT remains the gap between proof of concept and clinical validation. Robust development includes temporal or geographic data splitting, an independent external cohort, a preregistered analysis, and comparison with simple clinical models. Confidence intervals, calibration, the proportion of missing data, and the imputation procedure should be reported. For chemistry-focused models, scaffold splitting is additionally required, and for images, patient-level splitting. A complex architecture does not compensate for a small sample size and the lack of external validation [15,17,26].

Clinical utility is determined by whether the prediction changes the decision and improves the outcome. For this reason, promising systems should be evaluated in prospective studies as decision-support tools for choosing a photosensitizer, a light regimen, the need for fractionation, additional immunotherapy, or early repeat intervention. Net benefit analysis, assessment of the human factor, and recording cases in which the clinician rejects the algorithm’s recommendation are important. The ultimate aim is not to replace the specialist automatically, but to reproducibly integrate molecular, imaging, dosimetric, and clinical data into a personalized prediction with explicitly stated uncertainty [15,17,178].

Calibration should be assessed not only globally, but also in clinically meaningful subgroups: by tumor type, photosensitizer, center, skin phototype, and irradiation regimen. The same overall Brier score may conceal systematic overestimation of risk in one group and underestimation in another. When choosing a decision threshold, the cost of errors should be taken into account: missing a resistant lesion and unnecessarily intensifying therapy have different consequences. Therefore, the classification threshold should be set in advance on the basis of the clinical task, rather than tuned after looking at the test sample [26,178].

### 8.9. Chapter Conclusions

Predicting PDT outcomes should be framed as a multi-level task linking the photochemical dose to cell death, tissue response, immune remodeling, and the long-term clinical outcome. The most informative models will be multimodal and dynamic: they will combine baseline clinical features, imaging, omics signatures, photosensitizer distribution, and treatment-time monitoring data. At the same time, data quality remains the priority, because increasing the number of modalities without standardization raises the risk of systematic error. A practically useful model should be externally validated, calibrated, interpreted at the level of modifiable factors, and able to indicate when its prediction is unreliable [26,162,165,178].

At the near-term stage, more realistic than universal models for all PDT variants are specialized tools for a specific indication, photosensitizer, and equipment. Such models are easier to validate and integrate into the workflow. As standardized data accumulate, a transition to transferable representations and federated learning across centers may become possible, but only while preserving a transparent scope of applicability and the possibility of local recalibration [15,17,26].

Models and candidate biomarkers for predicting photodynamic therapy outcomes are systematized in Table A6.

## 9. AI-Guided Combined Photodynamic Therapy

### 9.1. The Concept of Rational Combination and the Role of Artificial Intelligence

Combined PDT is based on the assumption that the spatially and temporally limited generation of reactive oxygen species can be complemented by interventions that affect independent or compensatory mechanisms of tumor resistance. However, mechanically combining two effective methods does not guarantee synergy. The outcome is determined by the doses, sequence of administration, interval between exposures, oxygen status, subcellular localization of the photosensitizer, pharmacokinetics of the second agent, and the state of the tumor microenvironment. Therefore, the space of potential protocols quickly becomes combinatorial, and empirical trial-and-error proves economically and ethically inefficient. Artificial intelligence can rank combinations, assess probable synergy and toxicity, and select regimens that maximize the therapeutic window rather than merely the magnitude of the cytotoxic effect [14,179,180].

In this section, studies are considered evidence of AI-guided combination PDT only when they include explicit AI-derived input features, a computational combination-selection or optimization algorithm, and experimental validation involving PDT. Studies demonstrating biological synergy between PDT and another treatment without an AI-guided selection step are treated only as biological background and not as evidence of AI–PDT.

In computational terms, the task can be formulated as predicting the response function for the triad “photosensitizer or PDT mode–adjunct intervention–biological context”. The input data comprise chemical structures, molecular targets, transcriptomic and mutational profiles of cells, light parameters, oxygenation indicators, and experimental dose–response matrices. Graph neural networks describe structural and target-related relationships between agents; multimodal models combine chemical, omics, and phenotypic features; and Bayesian optimization and reinforcement learning are applicable to the sequential selection of new experiments [181,182,183]. For PDT, it is essential to include light as an independent variable: the same drug combination, under a different fluence rate or drug–light interval, can shift from synergy to antagonism.

### 9.2. Combining PDT with Chemotherapy and Molecularly Targeted Drugs

Biologically, PDT may interact with chemotherapy and molecularly targeted drugs through oxidative damage, inhibition of repair pathways, modulation of hypoxia, and altered drug delivery [14,180,184]. These findings provide biological rationale for combination therapy but should not by themselves be regarded as evidence of AI-guided PDT.

AI models make it possible to view dose not as a single value but as a response surface. For each pair, it is necessary to predict not only Bliss, Loewe, has, or ZIP synergy, but also selectivity for normal cells. Modern methods are trained on high-throughput combination matrices, molecular fingerprints, and expression profiles; the most useful are models capable of transferring predictions to previously unexplored drugs or cell lines [63,181,182,183]. For a model to qualify as AI-guided PDT combination selection, PDT-specific variables such as photosensitizer properties, light dose, fluence rate, oxygenation, and photobleaching should be included explicitly, and the computationally selected combination should subsequently undergo experimental PDT validation.

Potential targets such as PI3K–AKT–mTOR, MAPK, HIF-1, NRF2, VEGF, BCL-2, and autophagy pathways constitute the relevant biological background [179,180,185]. They should be presented as evidence of AI–PDT only when AI is actually used to select the target or regimen, and the selected combination is experimentally validated with PDT.

### 9.3. PDT and Immunotherapy

The immunogenic effects of PDT and its potential synergy with checkpoint blockade, STING agonists, cytokines, and vaccine strategies provide biological justification for combination approaches [186,187,188,189]. However, such studies should not be classified as AI-guided combination PDT unless AI is used for patient or regimen selection and the resulting strategy is experimentally validated in a PDT setting.

AI can solve two interrelated tasks: selecting the patient in whom PDT will create sufficient immune priming and choosing the optimal immune intervention. Input features include the spatial density of CD8^+^ cells, checkpoint expression, the interferon signature, mutation burden, the antigen-presenting apparatus, the cytokine profile, and the degree of hypoxia. A multimodal model that combines digital pathology, single-cell RNA sequencing, and PDT parameters may potentially distinguish “cold” tumors that can become immunologically active from tumors with persistent myeloid suppression [186,187,188]. Such modeling should be described as a prospective AI–PDT framework unless the cited study includes an explicit computational selection algorithm and experimental PDT validation.

Temporal optimization is critical. Checkpoint blockade before PDT may alter the composition of the infiltrate, whereas administration after PDT takes advantage of antigens and activated dendritic cells that have already been released. Reinforcement learning or dynamic Bayesian models can update decisions based on early markers of inflammation, photobleaching, perfusion, and circulating immune parameters. At the same time, the objective function must include the risk of immune toxicity and damage to normal tissue, not only the probability of a complete response [187,188,189].

### 9.4. Combining Photodynamic, Photothermal, and Sonodynamic Therapies

PDT–PTT combinations provide a biological and physical rationale for joint optimization of photochemical and thermal effects [172,180,190]. Unless the cited work includes AI-based regimen selection followed by experimental PDT validation, it should be presented as background rather than as direct evidence of AI-guided PDT combination therapy.

Similarly, PDT–SDT combinations represent a promising optimization problem, but should be counted as AI–PDT evidence only when an AI algorithm actually selects or optimizes the combined regimen, and experimental PDT validation is provided [191,192].

### 9.5. Potentiation of PDT Through Ferroptosis and Redox Modulation

Ferroptosis and redox modulation provide mechanistic background for PDT combinations involving iron, GPX4 inhibition, GSH depletion, or lipid metabolism modulation [193,194,195]. These studies should not be treated as evidence of AI-guided combination therapy unless AI explicitly informs combination selection, and the proposed regimen is validated experimentally with PDT.

AI-based prediction of ferroptotic vulnerability is a potential future strategy; however, it should be presented as prospective unless the model has been used to select a PDT combination that was subsequently validated experimentally.

### 9.6. Gas Therapy, Oxygen-Generating Systems, and Microenvironment Modulation

Gas therapy, oxygen-generating materials, catalase-like nanozymes, and oxygen carriers provide biological approaches for modifying the PDT microenvironment [15,180,196]. Unless these strategies are selected or optimized by AI and experimentally validated in PDT, they should be discussed as biological background rather than direct AI–PDT evidence.

AI could potentially integrate photoacoustic or BOLD imaging, perfusion, hypoxia-related gene expression, and photobleaching kinetics to support selection among oxygen-delivery or oxygen-conservation strategies. Importantly, a local increase in oxygen content is not always beneficial: it may intensify oxidative damage to normal tissue or alter the immune response. The model must therefore incorporate safety constraints and measurement uncertainty [15,196].

### 9.7. Assessment of Synergy, Causality, and Experimental Validation

The quantitative assessment of combinations depends on the chosen null model. Bliss independence assumes independence of action, Loewe additivity compares agents with interchangeable doses, HSA uses the best single effect, and ZIP combines the concepts of independence and similarity. The very same experiment can be classified in different ways, particularly in the case of steep or incomplete dose–response curves. For PDT, an additional problem is that the “dose” comprises light, a photosensitizer, and oxygen. Consequently, a correct matrix must include at least two pharmacological dimensions and irradiation parameters [15,181,182,183].

High cross-validation accuracy does not guarantee generalizability. Data leakage occurs when structurally similar compounds, repeated measurements of the same cell line, or images from the same animal appear in different samples. Scaffold splitting, leave-drug-out, leave-cell-line-out, and external validation using independent laboratory data are required. Promising predictions must be validated using complete dose–response matrices, multiple tumor models, analysis of normal cells, and mechanisms of action. Only studies in which computational ranking is followed by experimental validation of a PDT-containing combination should be considered direct evidence of AI-guided combination PDT [63,181,182,183,184].

The most mature concept for combined PDT is a closed-loop active learning cycle. The algorithm selects combinations with high expected utility or informativeness; the robotic platform measures viability, ROS, type of cell death, immune markers, and toxicity; the results are fed back into the model. Multi-criteria Pareto optimization makes it possible to simultaneously maximize the antitumor effect, minimize dark and light toxicity, and reduce the doses of the components. Such a system should not produce a single “best” solution, but rather a set of alternatives with confidence intervals and an explanation of key characteristics [15,63,182,183].

#### 9.7.1. Multi-Level Optimization of Sequence and Dosage

In combined PDT, the sequence of interventions is an independent determinant of the outcome. Prior administration of a target inhibitor may suppress antioxidant defenses prior to ROS generation, whilst the same drug, when administered after irradiation, may block the recovery of cells that have survived. Similarly, an immune adjuvant administered too early may not coincide with the peak release of DAMPs, whilst late administration of a cytotoxic agent may be limited by vascular stasis. These biological timing effects define the optimization space but are not themselves evidence of AI-guided PDT. Therefore, the algorithm must model not only the combination of components but also the timing strategy: the timing of administration, duration of exposure, the interval between fractions, and the possibility of re-treatment. Sequential models, Gaussian processes, and reinforcement learning, which utilize early biomarkers of response as feedback, are applicable to this task [63,181,182,183,184].

A clinically relevant model must take into account inter-patient variability in pharmacokinetics. The concentration of the photosensitizer in the tumor, the clearance rate of the second agent, liver and kidney function, plasma protein binding, and local perfusion determine the actual combined exposure. Population PK/PD models can be combined with machine learning to personalize the drug–light interval and the dose of the concomitant drug. However, the optimization must be robust: the recommended regimen must maintain acceptable efficacy under conditions of uncertainty regarding concentrations, optical parameters, and oxygen status, and not merely at nominal values [14,15,179,180].

#### 9.7.2. Explainability and Biological Validation of Computational Synergy

Explainability is particularly important when a model proposes a non-trivial combination. Attention weights or SHAP values do not, in themselves, prove the mechanism, but they may point to targets, pathways, or molecular markers that determine the prediction. These hypotheses should be tested by inhibiting or knocking out the putative target, analyzing ROS, lipid peroxidation, mitochondrial potential, and immunogenic markers, and conducting rescue experiments. Without such validation, high predictive accuracy remains associative and is poorly transferable between tumor models [182,183,185,193].

For clinically oriented ranking, it is useful to categorize evidence by levels:

Computational prediction → AI-guided combination selection → experimental PDT validation in vitro → organoid validation → in vivo efficacy and safety → prospective clinical validation. Each combination can be assigned a maturity and uncertainty rating, which prevents a promising hypothesis from being conflated with an established protocol. Multifunctional nanoplatforms, where pharmacokinetics, localization, release, photothermal effects, and immune activation all change simultaneously, should be rigorously evaluated; in such systems, the contribution of individual components must be deconvoluted using controls and ablation experiments [14,15,172,179,180,190,191,192,193,194,195,196].

### 9.8. Clinical Translation Prospects

The clinical application of AI in combined PDT requires a shift from cell lines to patient-specific models. Organoids, ex vivo tissue slices, single-cell models, and spatial omics may capture tumor heterogeneity more accurately than averaged profiles. When developing a plan, consideration should be given to pharmacological interactions, liver and kidney function, skin photosensitivity, concomitant therapy, and the availability of equipment. Adaptive protocols require prospective studies in which the algorithm’s recommendations are recorded prior to the outcome being known and compared with the standard approach [14,179,180,186].

In the short term, the most realistic option is not an autonomous system, but clinical decision support. It can rank several viable combinations, show the expected benefits and risks, indicate the level of evidence, and suggest monitoring parameters. Responsibility for the choice remains with the doctor, whilst the model must monitor domain shift and deterioration in calibration following a change in the photosensitizer, equipment, or patient population. Consequently, AI transforms combined PDT not by automatically searching for the most aggressive regimen, but through reproducible optimization of a causally grounded and safe therapeutic window [179,181,182,183]. The criterion for success should not be maximum cytotoxicity in the experiment, but rather a reproducible improvement in a clinically significant outcome, whilst maintaining controlled systemic and local toxicity, as confirmed by independent data. Furthermore, it is necessary to define, in advance, the clinical endpoint, the rules for discontinuing treatment, and the acceptable range of prediction uncertainty.

## 10. Clinical Translation and Specific Applications of Photodynamic Therapy

### 10.1. From an Experimental Platform to a Clinical Solution

The clinical translation of PDT is not a straightforward transfer of a successful “photosensitizer–light” combination from the laboratory, but rather a sequential alignment of chemical, optical, anatomical, and organizational factors. For different diseases, the therapeutic target may be located on the skin surface, in the mucous membrane, within a parenchymal organ, or in a bacterial biofilm; accordingly, the suitable photosensitizers, route of administration, depth of activation, endpoints, and risk profile vary. Recent reviews confirm the clinical efficacy of PDT for a range of oncological and non-oncological indications; however, they highlight the substantial heterogeneity of protocols and evidence [15,17,197,198]. In this context, artificial intelligence (AI) is most useful not as an autonomous “treatment choice”, but as an integrative layer: it is capable of integrating the molecular characteristics of a drug, imaging data, light delivery parameters, clinical phenotypes, and dynamic monitoring signals into an individualized intervention plan [15,17].

For each clinical indication below, four aspects are considered separately: the clinical maturity of PDT, the data types currently available for AI analysis, the present level of AI evidence and validation, and the most realistic near-term application. This distinction is necessary because established clinical use of PDT does not automatically imply that AI-guided patient selection, treatment planning, or dose adaptation has been clinically validated.

The practical readiness of AI–PDT systems should be assessed separately from the model’s accuracy on a retrospective sample. A clinically meaningful system must operate using data from a specific institution, take into account the available photosensitizer and light source, provide interpretable recommendations, assess uncertainty, and not disrupt the standard workflow. Therefore, the transition from proof-of-concept to clinical practice requires external validation, followed by prospective validation, testing of robustness against changes in equipment, analysis of the impact on the clinician’s decisions, and registration of the software component as a medical device. In the Clinical Readiness Level (CRL) scale proposed for this article, computational models without independent verification correspond to CRL levels 1–2; external validation and prospective observation correspond to CRL levels 3–4; only multicenter interventional trials and regulatory implementation provide grounds for classifying them as CRL levels 5–7 [15,16].

### 10.2. Dermatological Photodynamic Therapy

Clinical maturity: Dermatological PDT is an established treatment, particularly for actinic keratosis and selected superficial lesions [197,199,200]. Available data include clinical photographs, lesion dimensions, skin phototypes, fluorescence/PpIX imaging, hyperspectral data, and treatment parameters. Existing AI work is strongest in lesion segmentation, classification, and image-based assessment, whereas evidence for AI-guided PDT patient selection or adaptive dosing remains limited [15,199,200]. Validation is therefore mainly retrospective or proof-of-concept. The most realistic near-term application is standardized lesion mapping, documentation, and prediction of incomplete response or treatment tolerance.

AI can support dermatological PDT at three levels. Prior to the procedure, image segmentation and classification algorithms are capable of mapping the field of cancerization, assessing the area and heterogeneity of lesions, and identifying areas requiring a biopsy, rather than relying on empirical PDT. During treatment, the combination of RGB, hyperspectral, and fluorescence imaging potentially enables the prediction of PpIX accumulation and the local photodynamic dose. Following the procedure, models can assess the early inflammatory response, healing, cosmetic outcome, and the likelihood of an incomplete response. However, digital photography is dependent on lighting, the camera, and skin phototype; consequently, standardized color targets, calibration, and verification of model accuracy are required for different skin tones [15,199,200].

### 10.3. Head and Neck Tumours

Clinical maturity: PDT is used in selected superficial, early-stage, and recurrent head and neck lesions, but evidence is based on relatively small and heterogeneous cohorts [201,202,203,204]. Available data include CT, MRI, endoscopic, and fluorescence images, tumor geometry, critical-structure locations, and treatment parameters. AI research primarily concerns segmentation, navigation, light-distribution modeling, and potentially functional outcome prediction [201,202,203,204,205]. Clinical AI validation remains limited. The most realistic near-term scenario is AI-assisted treatment planning and delineation of tumor and critical structures, rather than autonomous patient selection.

The task for AI here is naturally formulated as multi-criteria planning. The model must segment the tumor and critical structures using CT/MRI and endoscopic images, determine the required irradiation geometry, and assess the risk of underdosing deep regions and over-irradiation of normal tissues. In superficial treatment, computer vision can assist with applicator navigation and the registration of fluorescent boundaries. For more complex geometries, physics-informed models of light propagation are useful. An additional factor is the prognosis regarding functional outcome: the same oncological response may have different clinical significance depending on the site of the tumor and the patient’s baseline condition [201,202,203,204,205].

### 10.4. Interstitial PDT and Neuro-Oncology

Clinical maturity: Interstitial PDT has reached clinical use in selected deep tumors, including recurrent gliomas, but remains considerably less standardized than superficial PDT [206,207,208,209]. Available data include MRI/CT-derived geometry, diffuser coordinates, tissue optical properties, photosensitizer fluorescence, spectroscopy, and intra-procedural temperature or dosimetry measurements. AI research is particularly relevant to light-transport surrogates, diffuser placement, dose-field optimization, and digital twin concepts [206,207,208,209,210]. Current validation is mainly technical, retrospective, or preclinical. The most realistic near-term application is AI-assisted treatment planning and intra-procedural replanning rather than autonomous therapeutic control.

Interstitial PDT represents one of the most realistic scenarios for a digital twin. An individual model may include the three-dimensional geometry of the tumor, coordinates of diffusers, optical coefficients, vascular regions, the distribution of the photosensitizer, and power constraints. Fast neural network surrogates for Monte Carlo modeling make it possible to recalculate the dose field multiple times as the position or power of the fiber changes. Intra-procedural data from fluorescence, spectroscopy, and temperature sensors can update the model and detect deviations from the plan. At the same time, the algorithm must not mask the uncertainty of the boundaries of an infiltrating tumor: in glioma, the area of microscopic spread often does not coincide with the contrast-enhanced volume visible on MRI, which requires robust safety margins and neurosurgical monitoring [206,207,208,209,210].

### 10.5. Gastrointestinal and Biliary Tumors

Clinical maturity: Endoscopic or intraluminal PDT has clinical experience in selected gastrointestinal and biliary malignancies, including unresectable cholangiocarcinoma [211,212]. Available data include endoscopic imaging, lesion and lumen geometry, stent anatomy, biochemical parameters, complications, and survival outcomes. Proposed AI applications include automatic lesion segmentation, lumen reconstruction, and light-distribution modeling, but validated AI–PDT studies remain scarce [15,211,212]. The most realistic near-term scenario is image-guided procedural planning and standardized outcome prediction.

An AI system for endoscopic PDT can combine automatic lesion segmentation, surface classification, lumen reconstruction, and light-distribution calculation. For biliary tumors, these data should be supplemented by biochemical parameters, infectious complications, stent anatomy, and previous interventions. A prognostic model should distinguish between technical success, local patency, symptomatic improvement, and overall survival, as these outcomes have different causal determinants. The small size of cohorts makes multicenter registries and temporal external validation particularly important, rather than the random splitting of data from a single center [15,211,212].

### 10.6. Ophthalmology

Clinical maturity: Verteporfin–PDT is clinically established for selected choroidal vascular disorders. Available data are relatively standardized and include OCT, OCT-angiography, fundus photography, fluorescein angiography, and indocyanine-green angiography [15,17]. AI research is comparatively mature for image segmentation and anatomical response assessment, but this should be distinguished from validated AI-guided selection of PDT regimen or dose. The most realistic near-term application is image-based treatment eligibility and response assessment using protocol-specific models.

### 10.7. Antimicrobial PDT

Clinical maturity: aPDT has been investigated clinically, particularly in dentistry, endodontics, peri-implant disease, and wound applications, but protocols and endpoints remain heterogeneous [64,213,214]. Available data include microbiological identification, biofilm characteristics, wound images, photosensitizer concentration, incubation time, wavelength, irradiance, fluence, and microbial log reduction. Direct AI-aPDT evidence is currently limited and predominantly conceptual [64,213,214]. The most realistic near-term goal is standardized data collection and prediction of microbial reduction rather than autonomous selection of treatments.

AI can personalize aPDT based on the type of microorganism, resistance profile, biofilm architecture, and local anatomy. This requires correlated datasets, including microbiological identification, photosensitizer concentration, incubation time, wavelength, irradiance, fluence, and log reduction in viability. Computer vision is capable of quantifying the wound area and dynamics of granulation, whilst spectral models can distinguish between the tissue, exudate, and bacterial load. However, current publications on the integration of AI and aPDT are predominantly conceptual; there are few clinically validated models. Therefore, the most realistic immediate objective is the standardization of data and the prediction of the likelihood of sufficient microbial reduction, rather than the autonomous replacement of microbiological testing [64,213,214].

### 10.8. Comparative Clinical Readiness and Translation Priorities

Across indications, the level of AI validation remains lower than the clinical maturity of PDT itself. Dermatology and ophthalmology offer relatively standardized imaging data; interstitial PDT provides a strong use case for physics-informed treatment planning; head and neck and gastrointestinal applications remain constrained by small, heterogeneous cohorts; and aPDT requires greater endpoint harmonization. Therefore, near-term translation should focus on indication-specific decision-support tools with external and prospective validation rather than a universal AI–PDT system [15,16,17].

Therefore, AI does not eliminate the biophysical limitations of PDT, but it can help to quantify them and ensure they are taken into account more consistently during treatment planning. A clinically useful system must answer specific questions: where to position the light source, which area requires an additional dose, which patient is expected to respond, and when to stop irradiation or opt for an alternative treatment. The most promising approach is not a universal model for all indications, but rather a modular architecture in which common components—data standardization, uncertainty assessment, and physics-informed modeling—are combined with disease-specific images and endpoints. This strategy ensures a gradual transition from computational demonstration to prospectively validated, safe, and reproducible personalized PDT [15,17,208].

### 10.9. Patient Selection and Integration into the Clinical Workflow

Patient selection models should integrate lesion characteristics, accessibility, photosensitizer accumulation, hypoxia, procedural risks, and relevant alternatives to PDT. Their clinical value should be demonstrated prospectively rather than inferred solely from retrospective discrimination metrics [15,16,17,197,198].

Integration into the workflow requires a division of responsibilities between the algorithm and the specialist. The system can automatically extract parameters from images, calculate lighting scenarios, and alert the user if the image falls outside the training domain, whilst the doctor confirms the diagnosis, selects the therapeutic goal, and makes the final decision. The safest mode is the “human-in-the-loop” mode, in which every change to the recommended power, the position of the light guide, or the duration of exposure is accompanied by an explanation and an assessment of the uncertainty. The interface should display clinically interpreted values rather than abstract probabilities: the prognosis for the underdosed zone, the risk of damage to a critical structure, the expected range of local control, and the factors that most influence the recommendation [15,16,17].

### 10.10. Multicenter Data and Post-Registration Surveillance

Multicenter AI–PDT datasets should contain harmonized information on the photosensitizer, administration protocol, drug–light interval, wavelength, fluence and fluence rate, delivery geometry, optical properties, photobleaching, oxygenation, imaging, toxicity, and standardized clinical endpoints. Validation should be performed across centers rather than after random pooling of observations [15,17,197,198].

Even after successful prospective research, the model’s performance may decline as a result of updates to cameras, light systems, photosensitizers, or changes in the patient population. Therefore, AI–PDT requires post-registration surveillance: monitoring of calibration, the frequency of clinician overrides of the recommendations, adverse events, and input data distribution drift. Model updates must follow a predefined protocol that includes version control, independent validation, and the option to revert to the previous algorithm. Federated learning can facilitate the use of multicenter data without the transfer of identifiable information, but it does not eliminate the need to harmonize annotation and to control systematic differences between institutions. It is precisely this infrastructure that transforms a single, high-precision model into a clinically supported technology [15,16,17].

## 11. Limitations, Data Standards, Ethics, and Regulation of Artificial Intelligence Systems in Photodynamic Therapy

### 11.1. From High Accuracy to Evidence-Based Clinical Reliability

Clinical reliability of AI–PDT depends primarily on data quality, protocol reproducibility, calibration, and external validation rather than internal accuracy alone. TRIPOD+AI and PROBAST+AI provide frameworks for transparent reporting and assessment of bias [26,27]. For PDT, reporting should additionally include the photosensitizer, formulation, light spectrum and geometry, fluence, fluence rate, drug–light interval, oxygenation, and response assessment methods.

AI–PDT evidence should distinguish analytical validity, clinical validity, and clinical utility. FUTURE-AI, DECIDE-AI, SPIRIT-AI, and CONSORT-AI provide complementary guidance for responsible development and clinical evaluation [28,32,215].

### 11.2. Limited and Heterogeneous Datasets

AI–PDT datasets are highly heterogeneous across chemistry, cell experiments, animal studies, optical systems, and clinical practice. Differences in photosensitizer formulation, experimental conditions, equipment, and endpoint definitions can prevent meaningful pooling and external validation. Data provenance, equipment, and preprocessing should therefore be documented in a machine-readable form [216,217].

A small effective sample size is particularly dangerous. Thousands of images may originate from just a few dozen patients, whilst hundreds of cells may come from a single microscopic field or a single experimental batch. Randomly splitting such objects between the training and test sets causes data leakage and inflates the metrics. The split should be carried out at the level of the patient, the animal, an independent experiment, or a chemical scaffold. For temporal models, chronological validation should be used, whilst for the generative design of photosensitizers, scaffold splitting and analysis of the distance to training structures should be employed. The prevalence of a high risk of bias in medical ML studies shows that the mere presence of a test sample does not compensate for an incorrectly defined unit of independence, optimistic feature selection, and the lack of calibration assessments [27,216].

### 11.3. Minimum Data Standard for AI–PDT

For molecular datasets, the following are mandatory: canonical SMILES or InChI, charge and protonation, metal coordination where applicable, including central or peripheral metal-containing motifs, solvent, pH, temperature, concentration, aggregation state, and measurement method. This distinction is necessary because metal-containing photosensitizers are not universally characterized by a centrally coordinated metal; in some molecular classes, the relevant metal-containing functionality may be peripheral. Spectral and photochemical measurements should include uncertainty, the reference standard, and the conditions under which the quantum yields were determined. For biological experiments, it is necessary to record the cell line and its authentication, passage number, cell density, culture medium composition, photosensitizer dose, incubation duration, light parameters, oxygen levels, time point, and raw data. For clinical PDT, the minimum dataset must also include the diagnosis, disease stage, anatomical geometry, method of drug administration, device parameters, light distribution, adverse effects, and a predefined endpoint. Such a standard must comply with the FAIR principles: data must be discoverable, accessible under reasonable conditions, interoperable, and reusable [26,32].

Controlled terminology, standardized units, and retention of imaging and processing metadata are required. Model cards should document intended use, limitations, population characteristics, and failure conditions.

The recommended minimum reporting standard for AI–PDT studies is described in Table A7.

### 11.4. Dataset Shift, Drift, and External Validation

Dataset shift may result from changes in patients, photosensitizers, cameras, light sources, preprocessing, or treatment protocols. External validation should therefore include geographical, temporal, and technological variation and report results separately for clinically relevant centers and subgroups [218].

Once the system has been implemented, it is necessary to monitor input distribution, missing-data rates, calibration, the frequency of erroneous recommendations, and clinical outcomes. The alert threshold should be defined in advance. If drift is detected, the model must not be automatically retrained on new data without version control: an update may correct one error but worsen the outcome in another group. It is recommended to separate the monitoring workflow from the workflow for releasing a new version, to carry out shadow testing and repeat external validation, and to document any changes to the intended use. For adaptive systems, the permissible scope of modifications and the conditions for reverting to the previous version should be defined in advance.

### 11.5. Fairness and Biologically Relevant Subgroups

AI–PDT performance should be assessed in clinically and biologically relevant subgroups because factors such as skin pigmentation, age, comorbidity, anatomical site, and access to specialist equipment can affect both measurements and outcomes [219,220]. Fairness criteria should be linked to the clinical consequences of errors rather than to a single universal metric.

Bias may also arise from differences in data completeness and annotation quality. Subgroup-specific performance, confidence intervals, and clinically relevant error patterns should therefore be reported [221,222,223].

### 11.6. Interpretability, Uncertainty, and Expert Involvement

The explanation should be addressed to a specific user. A chemist would find structural fragments that affect λ_max_ or ΦΔ useful; a medical physicist would benefit from information on the contribution of optical properties and the position of the optical fiber; and a physician would find useful the factors that predict the response and the range of uncertainty. Heatmaps or SHAP plots do not guarantee causality and may be unstable with minor changes in the data. A systematic analysis of XAI in healthcare reveals the absence of a single standard for evaluating the quality of explanations and the rarity of studies that verify whether explanations in fact improve human decision-making [224]. Therefore, explainability should be tested in user studies: decision-making time, frequency of algorithm re-evaluation, understanding of limitations, and the ability to recognize a false recommendation.

The AI–PDT system must be able to refrain from responding when the data fall outside the applicable range, when image quality is low, or when uncertainty is high. Conformal prediction, ensembles, Bayesian approximations, and separate out-of-distribution detectors are useful. The interface should display not only the prediction itself, but also the confidence interval, the similarity of the case to the training sample, and the parameters that deviate most from the norm. The human retains the right to make the final decision; however, a formal statement “to assist the physician” is not sufficient: responsibilities must be defined, staff must be trained, a protocol for deviating from the recommendation must be established, and the algorithm’s impact on treatment must be documented [28,225].

### 11.7. Privacy and Federated Learning

Federated learning can support multicenter AI–PDT development without centralizing raw data but does not eliminate privacy risks. Secure aggregation, access controls, data minimization, and governance remain necessary [226,227].

Privacy protection should be proportional to the sensitivity of the data and should include an explicit threat model, particularly for genomic and high-dimensional imaging data [228,229].

### 11.8. Reproducibility of the Software and Experimental Pipeline

Reproducibility requires documentation of both the computational pipeline and the physical PDT protocol. Software versions, preprocessing, model parameters, and validation procedures should be recorded together with light-source calibration, spectrum, detector configuration, and other relevant experimental conditions.

### 11.9. Cybersecurity and Resilience to Malicious Attacks

Cybersecurity should be considered part of AI–PDT clinical safety. Systems should protect model and software integrity, maintain audit trails, and apply independent physical limits to critical treatment parameters such as fluence, fluence rate, and exposure time. A safe fallback mode should permit treatment according to a validated non-AI protocol.

### 11.10. Regulatory Status and Lifecycle Management

A system that merely sorts research molecules poses a different risk than a program that recommends a dose to a patient. When used for diagnosis, prognosis, or treatment management, it may be considered “software as a medical device” or a component of a medical device. The regulatory assessment must cover the intended purpose, clinical evidence, cybersecurity, human factors, quality management, and post-market surveillance. In the European Union, requirements for high-risk AI are integrated with the MDR/IVDR; for the developer, this means the need for a single technical file, data traceability, risk management, and human control [32,225].

Model updates should follow predefined change-control procedures with versioning, verification, and post-market monitoring of calibration, clinical errors, and unintended use.

### 11.11. Generative AI, Research Integrity, and Practical Recommendations

Generative models can accelerate literature searches, the retrieval of photochemical parameters, and the generation of photosensitizer candidates; however, they carry the risk of fabricated references, incorrect DOIs, and plausible but chemically impossible structures. Therefore, every statement and bibliographic entry must be verified against the original source, and the proposed molecules must undergo validation of valence, charge, synthetic feasibility, and quantum-chemical parameters. Generative AI should not provide unverified clinical recommendations; when used as an interface to validated systems, its sources and model version should remain traceable.

Responsible AI–PDT development therefore requires predefined clinical questions, independent validation, calibration, preservation of PDT-specific metadata, defined applicability limits, appropriate data protection, and controlled lifecycle management.

Information about major barriers to AI–PDT and the proposed strategies to address them is systematized in Table A8.

## 12. Development Prospects and Conclusions

### 12.1. Fundamental Models for Photochemistry and Molecular Design

The next stage of the digital transformation of photodynamic therapy will be linked not so much to an increase in the number of individual algorithms as to the emergence of fundamental models capable of forming universal representations of chemical structure, spatial conformation, electronic dynamics, and biological context. Unlike traditional QSAR models trained on a single endpoint, such systems are first trained on large datasets of molecules, reactions, molecular dynamics trajectories, and scientific texts, and then adapted to relatively small photochemical datasets. The VideoMol model has demonstrated that using time information from molecular “videos” makes it possible to encode not only static structure but also dynamic transitions between conformations, which is fundamentally important for predicting the accessibility of excited states and intersystem conversion [230,231]. Generative diffusion models, which operate directly in three-dimensional space and take into account the geometry of the binding pocket, demonstrate the ability to perform a targeted search for structures with specified complementarity and optimized properties [232,233].

For PDT, the fundamental model must integrate several levels of description: the molecular structure of the photosensitizer, 3D conformations, quantum-chemical descriptors, absorption and emission spectra, the quantum yield of singlet oxygen, aggregation parameters, solvent, pH, data on subcellular localization, and phototoxicity results. Simply scaling existing drug design models is insufficient, since the photosensitizer acts through an excited state, and its properties depend significantly on the microenvironment. A prospective architecture involves supplementing a pre-trained molecular representation with physical constraints and specialized heads for predicting λ_max_, ΔEST, spin–orbit coupling, ΦΔ, photostability, and dark toxicity. The integration of machine learning with molecular modeling is already considered a key approach to designing nanocarrier systems; however, for AI–PDT, such integration must extend to the entire “photosensitizer–carrier–biotarget” complex [234].

### 12.2. Autonomous Discovery of Photosensitizers and Closed Experimental Cycles

The most transformative approach could be an autonomous laboratory in which a generative model proposes candidates, a synthetic feasibility algorithm selects feasible routes, a robotic platform performs synthesis and purification, and a spectroscopic and cellular unit automatically measures properties. The results are returned to the model via active learning or Bayesian optimization. Such a cycle is capable of transitioning from passive prediction to the sequential selection of experiments that maximize informational value. At the same time, the objective function should not be reduced to a single metric, such as ΦΔ. Multi-criteria optimization is required, which simultaneously takes into account red or near-infrared absorption, solubility, aggregation-induced changes, photobleaching, selectivity toward tumor cells, organelle targeting, synthetic complexity, and safety [232,233].

The practical value of autonomous search will be determined by the quality of the feedback. Measurements must be conducted under standardized conditions, and the model must receive not only successful but also negative results. It is significant to assess uncertainty and the ability to identify candidates that fall outside the scope of applicability. For compounds with a central metal, the coordination environment, charge, spin state, and possible photochemical transformations must be explicitly encoded. In the future, an automated platform should incorporate DFT calculations as an intermediate filter, but not replace experimentation: predictions of excited states are sensitive to the chosen functional, solvent, and conformation. Therefore, the most realistic approach is a multi-stage system in which low-cost ML screening narrows down the search space, high-precision calculations refine the top candidates, and physical experiments confirm the photophysical and biological characteristics.

### 12.3. Digital Twins of the Patient and Tumor

A digital twin in AI–PDT should be understood as a dynamic computational model of an individual patient that is continuously updated as new data become available and enables alternative treatment regimens to be compared before they are implemented. The current concept of medical digital twins integrates mechanistic models, statistical learning, and continuous measurements; however, its clinical maturity remains limited [235,236]. For PDT, a digital twin should incorporate the three-dimensional geometry of the tumor and critical structures, maps of light absorption and scattering, vascular perfusion, oxygenation status, photosensitizer distribution, and photobleaching kinetics. At the biological level, it must take into account the sensitivity of cell subpopulations to ROS, antioxidant potential, the likelihood of vascular damage, and immunogenic cell death.

The concept of a theranostic digital twin, developed for personalized radiopharmaceutical therapy, provides a useful framework for PDT as well: diagnostic imaging and biomarkers are used to initialize the model, treatment is represented as a controlled intervention, and post-treatment measurements are used to update the twin’s parameters [237]. In oncology, digital twins have also been proposed for optimizing treatment regimens, virtually comparing therapeutic strategies, and designing more efficient clinical trials [238,239]. However, translating this concept into photomedicine requires high-frequency data acquisition during the procedure. Photosensitizer fluorescence, diffuse reflectance, oxygenation, temperature, and blood flow should be fed into the model with minimal delay. Under these conditions, the digital twin could not only predict treatment outcomes but also recommend adjustments to light power, fractionation, or termination of irradiation.

A major scientific challenge remains parameter identifiability. Several different combinations of photosensitizer concentration, oxygen concentration, and optical properties may produce similar observable signals. Therefore, the digital twin should provide a distribution over possible system states rather than a single point estimate. Bayesian methods, ensemble approaches, and sequential data assimilation will be required. The clinical system should clearly distinguish between measured parameters, model assumptions, and sources of uncertainty, and communicate this uncertainty to the clinician. Otherwise, the digital twin risks becoming a sophisticated visualization tool that does not improve the safety of clinical decision-making.

### 12.4. Adaptive Dosimetry and Real-Time PDT Control

The personalized PDT of the future must transition from a predetermined light exposure to closed-loop control. In such a system, a model assesses the current photodynamic state of the tissue and selects the next action: continue irradiation, reduce the fluence rate, pause for reoxygenation, adjust the power distribution among the light guides, or terminate the procedure. It is fundamentally important to optimize not the nominal fluence, but the biologically effective dose, which is determined by the combined action of light, the photosensitizer, and oxygen. This requires fast surrogate models of light transport and photochemical kinetics, which are trained on physical simulations but calibrated with real patient signals.

Fundamental models and digital twins should not completely replace mechanistic descriptions. On the contrary, the most reliable architecture is a hybrid system: a physical model constrains the space of feasible solutions, while ML compensates for unknown parameters, systematic errors, and individual differences. This approach reduces the risk of implausible recommendations due to domain shift. For clinical safety, an adaptive controller must have strict limits on power, temperature, time, and dose, as well as a manual confirmation mode. In the early stages of implementation, it is advisable to use AI as an advisory system, comparing its recommendations with those of a specialist, rather than granting the algorithm autonomous control.

### 12.5. Multicenter and Federated Learning

The transition from pilot models to reproducible clinical algorithms is impossible without multicenter data. PDT is used in various clinical settings, with different photosensitizers, light sources, and protocols; therefore, a single-center model almost inevitably reflects local characteristics of equipment and patient selection. Federated learning enables the training of a shared model without the centralized transfer of raw data and is already being actively researched in medicine and computational pathology [240,241,242]. It is particularly promising for AI–PDT, as images, spectra, and genomic data may be subject to strict confidentiality restrictions.

However, federated learning alone does not eliminate heterogeneity. Differences in wavelength, detector calibration, image resolution, diagnostic criteria, and endpoints result in non-IID distributions. Therefore, future consortia must agree on a minimum set of PDT metadata, units of measurement, calibration methods, and rules for determining responses. Promising approaches include personalized federated models, where the common component studies universal patterns, while local components adapt to the center’s equipment and population. Secure aggregation, audit of updates, verification of resistance to malicious participants, and uncertainty assessment at each site are also required.

### 12.6. Causal AI, Multimodality, and Individual Treatment Effects

Most modern AI models predict the probability of response but do not answer the clinically more important question: will the outcome change if a different photosensitizer, drug–light interval, or irradiation regimen is chosen for a specific patient? Causal machine learning provides tools for modeling interventions and the heterogeneity of therapeutic response [243,244]. In AI–PDT, this will enable a shift from risk stratification to action selection. For instance, a high risk of incomplete response does not mean that a patient should not receive PDT; perhaps the risk is reduced with fractionated irradiation or an oxygen-modulated combination.

Assessing the individual therapeutic effect requires sufficient overlap among treatment options and proper control of confounding factors. In observational data, the choice of protocol is related to tumor size, location, the center’s experience, and the availability of equipment. Therefore, causal models must be accompanied by a predefined causal graph, sensitivity analysis, and, where possible, validation in randomized or pragmatic trials. Methods for assessing individualized treatment effects are already being developed for health technology assessment and can be adapted to multiparametric PDT protocols [245].

Accurate personalization requires the multimodal integration of molecular, imaging, spectroscopic, and clinical data. Representations must preserve temporal and spatial structure: the average concentration of a photosensitizer or the average gene expression value does not reflect tumor heterogeneity. Graph and multiview models allow for the integration of mutations, protein interactions, and tissue architecture while preserving interpretable components [246]. In the future, the model should explain which data points led to a change in the recommendation and present a counterfactual scenario: how the prognosis would change with a different dose or light-guide location.

### 12.7. Translational Roadmap and Final Conclusions

**Figure 7:**
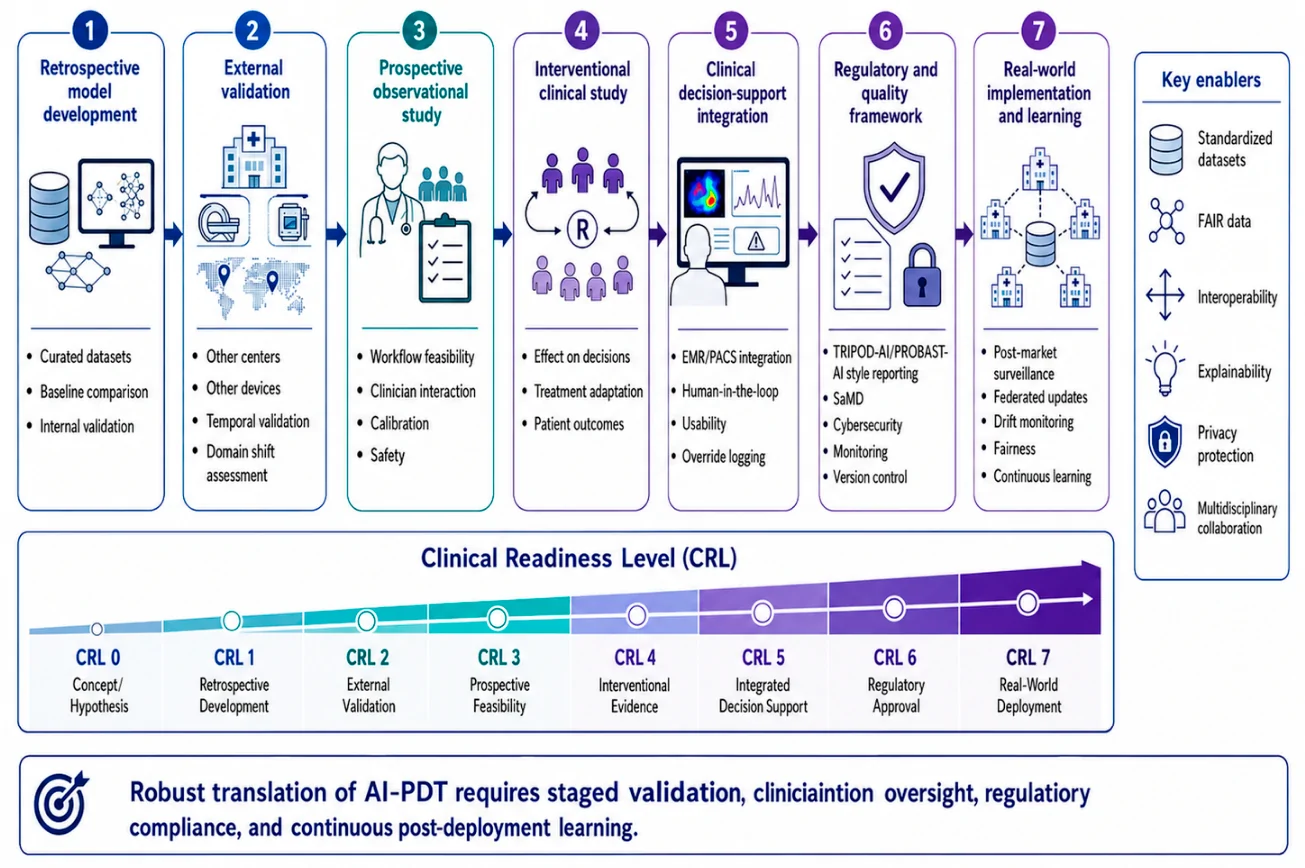
Translation roadmap for AI-enabled PDT.

The realistic translation of AI–PDT requires a step-by-step roadmap. The first level consists of retrospective studies with transparent data separation and comparison to clinical baseline models. The second level involves external validation at an independent center and calibration analysis. The third level involves prospective observational use, in which the model’s recommendations are recorded but do not influence treatment. The fourth level involves an interventional study assessing clinical benefit, safety, and impact on workflow. Regulated implementation, drift monitoring, and controlled updates are possible only thereafter. The general limitations of medical AI—systematic biases, lack of transparency, reduced quality outside the training environment, and overreliance on automation—fully apply to PDT as well [79]. The translational roadmap of AI-driven PDT is shown in Figure 7.

A key prerequisite for progress is a change in publication culture. In addition to ROC–AUC, calibration, uncertainty, error characteristics, and clinical utility should be reported. For molecular models, scaffold-split analysis and experimental validation of new chemical classes are required; for images, separation at the patient and center levels; and for digital twins, validation of each mechanistic module and the entire system. Code, data dictionaries, photosensitizer versions, light parameters, and negative results should be made available to the extent permitted by ethics and intellectual property considerations. Immune digital twins emphasize that a personalized model must incorporate the dynamics of the immune system, not just the tumor mass [247,248,249].

Ultimately, artificial intelligence is capable of transforming PDT not as an isolated analytical tool, but as an integrative infrastructure. At the molecular level, it accelerates the design of photosensitizers and carriers; at the biophysical level, it integrates light transport, oxygen, and photodegradation; at the cellular and tissue levels, it interprets images, omics profiles, and cell death types; and, at the clinical level, it assesses individual efficacy, toxicity, and the optimal protocol. The most promising approach is a closed-loop system: molecular design → delivery → planning → monitoring → outcome → feedback. Its implementation must be grounded in causality, physical constraints, multicenter validation, and the preservation of the physician’s leading role. Only under these conditions will AI–PDT be able to transition from a set of promising proof-of-concept solutions to reproducible, personalized photomedicine.

Therefore, the immediate goal of the field is not to create the most complex neural network possible, but to build a verifiable ecosystem of data and models. Fundamental molecular models, autonomous laboratories, digital twins, adaptive dosimetry, federated learning, and causal analysis are complementary components of this ecosystem [79,231,235,236,237,238,239,240,241,242,243,244,245,246,247]. Combining these elements will make it possible to select a photosensitizer and carrier based on the patient’s biology, model the spatial photodynamic dose, adjust treatment based on real-time signals, and use the results to improve future decisions. This approach preserves the central role of photochemistry and biophysics while transforming accumulated data into clinically applicable knowledge.

A separate area of focus should be the development of prospective benchmark datasets designed not only for training but also for the comparative evaluation of algorithms. Such a dataset should include raw spectra and images, segmentation masks, time series of photobleaching and oxygenation, light source parameters, the chemical identity and batch of the photosensitizer, the clinical endpoint, and information about the center. In order to prevent overfitting, the closed test set must be stored by an independent coordinator, and evaluation must be performed at the patient and clinical center levels. Such infrastructure will make it possible to distinguish genuine methodological progress from improvements attributable to the characteristics of a single dataset. An important addition would be “worst-case scenario” modeling: low signal, missed time points, equipment changes, unusual anatomy, and rare adverse reactions. The results of such stress tests should influence the system’s level of clinical readiness.

Equally important is the issue of economic and organizational efficiency. Even an accurate model will not be implemented if it requires time-consuming manual data entry, complex calibration, or infrastructure that is inaccessible to most centers. Therefore, future studies should compare not only biological efficacy but also procedure duration, staff workload, sensor cost, the number of repeat interventions, and the patient’s quality of life. An optimal AI component can be relatively simple if it consistently reduces dosimetric variability and prevents incomplete treatment. In contrast, a complex digital twin without a clear clinical impact has no intrinsic value. Translational evaluation must consider the interaction between the clinician and the algorithm: how quickly the clinician understands the recommendation, recognizes an error, and can safely override the decision. It is the combination of scientific validity, usability, and measurable clinical benefit that will determine the sustainability of AI–PDT.

Finally, the field’s advancement requires interdisciplinary teams in which photochemists, optical specialists, oncologists, bioinformaticians, engineers, and regulatory experts jointly define endpoints and error criteria. Without such collaboration, an algorithm may be technically flawless but biologically irrelevant or impractical for clinical use. Co-design should begin before data collection, not after the model has been trained.

## 13. Conclusions

Artificial intelligence is gradually evolving from a supplementary computational tool into one of the key drivers of systemic transformation in photodynamic therapy. Its significance is determined not only by the ability to automate individual stages of data analysis, but also by the capacity to integrate the molecular design of photosensitizers, the development of delivery systems, light distribution modeling, tissue oxygenation assessment, image interpretation, prediction of biological responses, and the personalization of clinical protocols into a single, closed-loop technological cycle. In such a system, the results of each stage can be used to adjust subsequent decisions, and the accumulated experimental and clinical data can be used to continuously improve the models.

At the molecular level, machine learning methods can significantly accelerate the search for new photosensitizers. Classical QSAR and QSPR approaches, graph neural networks, generative models, and active learning algorithms make it possible to predict spectral parameters, the quantum yield of singlet oxygen, solubility, lipophilicity, photostability, tendency to aggregate, dark toxicity, and potential subcellular localization. The integration of machine learning with quantum-chemical calculations paves the way for a more accurate analysis of energy levels, intercombination conversion, spin–orbit interaction, and the mechanisms of reactive oxygen species formation. However, the reliability of such models remains limited by small, heterogeneous, and poorly standardized datasets, which vary in terms of solvents, measurement methods, experimental conditions, and methods of representing molecular structures.

Another equally critical area is the optimization of photosensitizer delivery systems. Artificial intelligence can be used to select the composition of liposomes, polymer nanoparticles, micelles, hydrogels, organometallic scaffolds, protein carriers, and exosome-like platforms. It enables the modeling of particle size, polydispersity, ζ-potential, encapsulation efficiency, release rate, stability, cellular uptake, and interaction with the protein corona. A promising development is the shift from optimizing individual physicochemical characteristics to multiscale modeling that integrates carrier properties, vascular permeability, interstitial diffusion, cellular transport, endosomal yield, and organelle targeting. From a clinical perspective, this could lead to the development of formulations adapted not only to the tumor type but also to the individual characteristics of a specific patient’s microenvironment.

Artificial intelligence plays a particularly significant role in planning light exposure and dosimetry. The traditional approach, based primarily on the amount of light dose administered, does not reflect the actual biologically effective photodynamic load. The outcome of PDT depends on the photosensitizer concentration, light absorption and scattering, oxygenation, perfusion, photobleaching, the rate of reactive oxygen species generation, and the geometry of the irradiated tissue. Neural network surrogate models can accelerate light transport calculations, reconstruct tissue optical parameters, optimize the positioning of light guides, and predict spatial dose distribution. When combined with sensors for oxygen, fluorescence, temperature, and blood flow, such models form the basis for adaptive PDT, in which irradiation parameters are adjusted during the procedure in response to the current state of the tissue.

Computer vision and spectroscopic analysis expand the capabilities for monitoring PDT efficacy at the cellular, tissue, and clinical levels. Deep learning algorithms can identify signs of damage, assess photobleaching, classify types of cell death, and detect early signs of therapeutic response. Cell and tumor segmentation should be considered separately as an image-analysis, diagnostic, or treatment-planning task rather than as direct evidence of PDT efficacy monitoring. Fluorescence imaging, FLIM, Raman spectroscopy, hyperspectral methods, OCT, and radiomics generate multidimensional data that cannot be fully interpreted using traditional statistical methods. Multimodal models that integrate images, spectra, clinical characteristics, omics data, and procedural parameters are of the greatest value. However, their practical application requires strict control of data bias, reproducibility of preprocessing, and model robustness to differences in equipment and clinical centers.

Outcome prediction is one of the most clinically significant areas of AI–PDT. Models can assess the probability of complete or partial response, local control, recurrence, toxicity, and survival. Clinical features, photosensitizer and light parameters, radiometric characteristics, transcriptomic signatures of hypoxia and oxidative stress, indicators of antioxidant defense, immune markers, and metabolic profiles are used for this purpose. However, high discriminatory power alone is not sufficient for a model. Reliable calibration, analysis of clinical utility, external validation, uncertainty assessment, and comparison with existing clinical algorithms are required.

Artificial intelligence has the potential to improve the design of combined PDT, including combinations with chemotherapy, immunotherapy, photothermal and sonodynamic therapy, ferroptosis-inducing strategies, and oxygen-generating systems; for most of these applications, the evidence remains preclinical at this time. Its potential role lies in selecting and optimizing combinations, doses, treatment sequences, and time intervals. However, studies demonstrating biological synergy alone should not be considered evidence of AI-guided combination PDT unless an AI-based selection or optimization step is followed by experimental validation involving PDT.

The main obstacles to clinical translation remain the lack of large, multicenter datasets, heterogeneity in protocols, the absence of uniform standards for reporting photochemical and clinical parameters, limited interpretability, the risk of data leaks, and weak external validation. To overcome these limitations, standardized AI–PDT datasets are needed, along with mandatory descriptions of experimental conditions, the separation of samples at the patient and independent series levels, the publication of code and preprocessing protocols, and the application of FAIR principles. Federated learning, uncertainty assessment, causal analysis, and prospective studies will play an important role.

One of the most significant—though as yet largely conceptual—outcomes of integrating artificial intelligence and photodynamic therapy could be the development of digital patient twins. Such systems will be able to combine the molecular properties of the photosensitizer, delivery parameters, three-dimensional anatomy, optical characteristics of tissues, oxygenation, the vascular network, imaging data, and the molecular profile. Such development will make it possible to simulate alternative treatment scenarios before the procedure begins and to adjust therapies in real time.

Thus, artificial intelligence does not replace the physical, chemical, biological, and clinical foundations of PDT, but rather provides a computational framework for their integration. Its greatest value lies in the transition from empirically standardized protocols to a mechanistically grounded, adaptive, and personalized photodynamic therapy. Realizing this potential will depend not so much on the complexity of the algorithms as on the quality of the data, experimental reproducibility, clinical validation, and close collaboration among chemists, photobiologists, clinicians, bioinformaticians, and artificial intelligence experts.

## Data Availability

No new data were created or analyzed in this study. Data sharing is not applicable to this article.

## Appendix A

### Supplementary Tables Supporting the AI–PDT Workflow

Appendix A Table A1, Table A2, Table A3, Table A4, Table A5, Table A6, Table A7 and Table A8.

**Table A1:** Artificial intelligence methods and their potential roles in the photodynamic therapy workflow.

Method Group	Main Role in AI–PDT	Key Value/Limitation
Classical statistical/chemometric models	Regression, PLS, and SVM; tabular, spectral, and clinical data	Baseline prediction, spectroscopy, and response modeling; interpretable but limited for complex nonlinear data [15,26,65,77,78,230]
Tree-based ensemble models	Random forest, XGBoost, CatBoost; mixed molecular, formulation, and clinical variables	Strong performance on structured data; risk of overfitting and domain-shift sensitivity [26,65,79]
Neural and temporal models	Fully connected networks, RNNs, and temporal models	Nonlinear prediction and longitudinal monitoring; higher data requirements and limited explainability [15,66,78,230]
Image-based deep learning	CNNs; microscopy, fluorescence, CT/MRI, OCT, and hyperspectral imaging	Segmentation, treatment-zone assessment, and response analysis; sensitive to imaging protocol differences [66,67,74,75]
Multimodal and graph-based models	Transformers and GNNs integrating molecular, imaging, omics, and clinical data	Integrated response prediction and molecular design; data-intensive and often retrospective/conceptual [15,68,69,70,79,80,231]
Generative and latent-space models	Autoencoders, VAEs, GANs, and diffusion models	De novo design, dimensionality reduction, and data augmentation; chemical feasibility requires validation [92,93,95,97,232,233]
Bayesian/active learning optimization	Gaussian processes and Bayesian optimization	Efficient selection of experiments, formulations, and PDT parameters under uncertainty [91,94,234]
Closed-loop and physics-informed AI	Reinforcement learning, PINNs, digital twins, and XAI	Adaptive control, physics-constrained planning, and interpretable decision support; mainly preclinical/pilot-stage [15,63,72,73,74,75,76,181,182,183,184,224,230,235,236,237,238,239,247]

**Table A2:** Machine learning models for predicting photosensitizer properties.

Task/Class	AI Approach	Main Conclusion
ΦΔ/photosensitizing efficiency	QSPR, tree ensembles, and ML using fingerprints, descriptors, and medium-specific variables	Good in-domain prediction; heterogeneous experimental conditions limit transferability [85,86,87,88,89,98]
BODIPY spectral/photochemical prediction	Regression, QSPR, and web-based ML tools	Useful for λabs/λem, ΦF, and ΦΔ screening; mostly computational or internally validated [86,87,96]
Transition-metal complexes	DFT-derived descriptors combined with ML	Accelerates screening of excited-state properties; synthesis and biological validation still required [90]
Large-scale virtual screening	Deep learning, Bayesian optimization, and active learning	Searches millions of candidates and prioritizes experimentally testable PSs [91,94]
Multi-objective/Type I design	ML-assisted optimization and iterative closed-loop discovery	Balances NIR absorption, ROS generation, and hypoxia tolerance; experimental validation available for selected candidates [92,93]
Aggregation-sensitive photophysics	Random forest/gradient boosting on monomeric and aggregated states	Captures AIE-related changes in quantum yield and wavelength [95]
General fluorophore datasets	Ensemble regression on literature-derived molecular datasets	Useful for preliminary spectral filtering but not sufficient to predict PDT efficacy directly [88,101]

**Table A3:** AI-oriented development of photosensitizer delivery systems.

Carrier Group	Optimization Focus	PDT Relevance
Lipid-based carriers	Liposomes, lipid nanoparticles; composition and manufacturing variables	AI can optimize size, PDI, loading, stability, and cellular delivery; PDT objectives must include photochemical activity and the drug–light interval [102,103,104,105,110,111,112,113,114,119,120,121,122,123]
Polymeric systems	PLGA nanoparticles, micelles, and nanogels	Multi-criteria optimization of size, loading, release, and aggregation; ROS generation after encapsulation must be validated [102,103,104,105,106,107,108,110,113,114]
Porous inorganic/framework carriers	Mesoporous silica, MOFs, and COFs	Joint optimization of loading, release, ROS/oxygen behavior, and stability; long-term clearance and dark toxicity remain important [102,103,104,105,106,107,109,110,115,116,117,118,123]
Biological carriers	Protein nanoparticles, albumin, exosomes, and membrane-coated particles	AI may predict biological identity, uptake, and batch variability; standardization remains difficult [103,104,105,106,109,115,116,117,118]
Targeted delivery systems	Ligand- and organelle-targeted nanoparticles	Models can integrate receptor expression, intracellular trafficking, and localization; targets should be measured directly [104,108,109,119,120,121,122,123]
Stimuli-responsive/process-aware systems	pH-, ROS-, hypoxia-, enzyme-, or light-responsive systems; microfluidic manufacturing	AI can optimize release kinetics and manufacturing conditions, but prospective validation and drift monitoring are needed [102,103,106,110,111,112,113,114,123]

**Table A4:** AI methods in PDT planning and dosimetry.

Application	Key Inputs	AI Contribution/Validation
Interstitial PDT	Patient geometry, optical properties, and fiber coordinates	ML/physics models enable optical property recovery, fiber power optimization, and near-real-time replanning; mainly simulation/preclinical [124]
Superficial PDT	Three-dimensional surface geometry, angle, distance, and irradiance	Geometric optimization improves illumination uniformity; pilot validation only [125,126]
Image-guided/intraoperative PDT	Pre-operative imaging, treatment geometry, and applicator parameters	Supports personalized target coverage while limiting normal-tissue exposure [18]
Brain/deep-tumor PDT	MRI/CT anatomy, diffuser placement, and optical parameters	Physics-informed planning and dose-field optimization are promising but clinically limited [127,128,129,130]
Real-time adaptive dosimetry	Fluorescence, oxygenation, photobleaching, and other dynamic signals	Supports intra-treatment dose adjustment; prospective clinical validation remains limited [131,132,133,134,135]
Digital twin/multimodal planning	Anatomy, optical, vascular, and biological information	Allows scenario comparison and adaptive control; currently conceptual/pilot-stage [235,236,237,238,239,247]

**Table A5:** AI-compatible monitoring across the PDT workflow.

Stage/Modality	What is Measured	AI Role/Caveat
Pre-treatment imaging	Lesion/tumor segmentation and baseline characterization	Diagnostic/treatment-planning task rather than PDT efficacy monitoring; used for target definition and planning [66,67,74,75]
Fluorescence/photobleaching	PpIX/PS intensity and bleaching kinetics during PDT	Tracks photosensitizer behavior and treatment dynamics; requires calibration and protocol standardization [15,66,230]
FLIM/spectroscopy	Lifetime, Raman, and diffuse reflectance features	Provides biochemical and microenvironmental information for intra-treatment assessment [77,78,230]
Hyperspectral/OCT/radiomics	Spatial tissue structure and optical signatures	Supports treatment-zone assessment and early response analysis; equipment; preprocessing strongly affect models [66,67,74,75]
Physiological monitoring	Oxygenation, perfusion, blood flow, and temperature	Enables adaptive intra-treatment control and detection of treatment-induced changes [15,66,230]
Post-treatment response	Follow-up imaging, tissue damage, residual disease, and outcomes	Used for early response and recurrence/outcome prediction; requires independent clinical validation [15,66,67,74,75]

**Table A6:** Predictors and biomarkers of PDT outcome.

Predictor Group	Examples	Relevance/Validation
Clinical/anatomical factors	Tumor size, stage, depth, site, and accessibility	Useful baseline predictors but insufficient without PDT-specific parameters [15,16,17,197,198]
Photosensitizer/dosimetry variables	PS dose, drug–light interval, fluence, fluence rate, and geometry	Directly relevant to response prediction and should be standardized across cohorts [15,16,17]
Optical/imaging biomarkers	Fluorescence, photobleaching, OCT, radiomics, and multimodal imaging	Potential predictors of early response and local control; external validation remains limited [66,67,74,75]
Hypoxia/redox biomarkers	Oxygenation, HIF-related signaling, and antioxidant status	Mechanistically relevant to PDT sensitivity; heterogeneous measurement methods limit pooling [15,179,180]
Omics/immune biomarkers	Transcriptomic, metabolic, and immune features	May improve individualized response prediction but most evidence is preclinical or retrospective [186,187,188,189]
Multimodal predictive models	Combined clinical, imaging, molecular, and treatment features	Highest potential for outcome prediction, but calibration, uncertainty, and external validation are essential [15,16,17,79]

**Table A7:** Minimum reporting standard for AI–PDT studies.

Reporting Domain	Minimum Information	Why It Matters
Molecular identity	SMILES/InChI, charge, protonation, metal coordination where applicable, concentration, and aggregation	Prevents models learning inconsistent chemical representations [26,32]
Photophysical conditions	Solvent, pH, temperature, wavelength, reference standard, and measurement method	Essential for λ_max_, ΦF, and ΦΔ comparability [26,32]
Biological experiment	Cell line/authentication, passage, density, medium, incubation, oxygenation, and time point	Reduces batch and protocol confounding [26,32]
Light/dosimetry	Light source, spectrum, fluence, fluence rate, geometry, and drug–light interval	Required to compare PDT dose and response across studies [26,32]
Imaging/spectral data	Raw data, acquisition settings, reconstruction/preprocessing, and masks	Supports reproducibility and prevents equipment-specific bias [26,32]
Clinical context	Diagnosis, stage, anatomy, treatment protocol, toxicity, and predefined endpoint	Needed for clinical validity and utility assessment [26,32]
Validation/reporting	Independent splits, calibration, uncertainty, subgroup metrics, and applicability domain	Prevents data leakage and overoptimistic performance estimates [26,27,32]
Lifecycle/governance	Model version, intended use, updates, drift, privacy, and audit trail	Supports safe multicenter and clinical implementation [28,32,215]

**Table A8:** Major barriers to AI–PDT and proposed strategies.

Barrier	Main Risk	Condensed Solution
Small, heterogeneous datasets	Overfitting and poor transferability	Standardized multicenter datasets, harmonized metadata, and external validation [27,216,217]
Data leakage/non-independent samples	Inflated performance estimates	Split at the patient, experiment, animal, or scaffold level [27,216]
Protocol and equipment variability	Dataset shift and calibration drift	Record device/protocol metadata; perform temporal, geographical, and technological validation [218]
Limited interpretability/uncertainty	Unsafe or overconfident recommendations	Use uncertainty estimates, applicability-domain checks, and human oversight [28,224,225]
Bias and subgroup imbalance	Unequal performance across populations	Representative data and subgroup-specific evaluation [219,220,221,222,223]
Privacy and multicenter data access	Restricted data sharing	Federated learning with secure aggregation and governance [226,227,228,229]
Cybersecurity/lifecycle risk	Manipulation, unsafe updates, and post-deployment degradation	Integrity checks, audit trails, change control, and safe fallback modes [32,225]
Weak clinical validation	Proof-of-concept does not demonstrate clinical utility	Prospective, multicenter, and interventional evaluation against clinical baselines [26,27,28,215]
